# Wide-range robust wireless power transfer using heterogeneously coupled and flippable neutrals in parity-time symmetry

**DOI:** 10.1126/sciadv.abo4610

**Published:** 2022-06-15

**Authors:** Hyunwoo Kim, Seungwon Yoo, Hyunwoo Joo, Jongheon Lee, Donggeun An, Seonghyeon Nam, Hyungu Han, Dae-Hyeong Kim, Sanghoek Kim

**Affiliations:** 1 Department of Electronic Engineering, Kyung Hee University, Yongin-si 17104, Republic of Korea.; 2 Center for Nanoparticle Research, Institute for Basic Science (IBS), Seoul 08826, Republic of Korea.; 3 School of Chemical and Biological Engineering, Institute of Chemical Processes, Seoul National University, Seoul 08826, Republic of Korea.; 4 Department of Electronics and Information Convergence Engineering, Kyung Hee University, Yongin-si 17104, Republic of Korea.; 5 Institute for Wearable Convergence Electronics, Kyung Hee University, Yongin-si 17104, Republic of Korea.; 6 Department of Materials Science and Engineering, Seoul National University, Seoul 08826, Republic of Korea.

## Abstract

Recently, stationary wireless power transfer (WPT) has been widely adopted in commercial devices. However, the current WPT configuration is limited in its operational area and susceptible to operating condition changes, impeding its applications for dynamic environments. To overcome the limitations, we propose a WPT system with laterally aligned neutral elements in parity-time (PT) symmetry, which can widen the operational area with the number of neutrals *N*. Compared to the conventional multiple-input–single-output WPT, the dimension of system complexity is substantially reduced from *R* × *C^N^* to *R*^*N+*1^ because the neutral amplitudes are simply controlled by coupling capacitors. The operational frequency is automatically adjusted to a real eigenvalue of the PT-symmetric system to achieve high voltage gain and efficiency, making the system robust. The performance of the system calculated by the coupled-mode theory was experimentally verified with rigid and flexible types of receivers, confirming its potential in both industrial and biomedical electronics.

## Teaser

Wireless power delivery using multiple neutrals to widen the operational area with minimal system complexity.

## INTRODUCTION

Recently, the radiofrequency (RF) wireless power transfer (WPT) method has attracted considerable interest because it serves as one of the key power supply methods in various emerging applications such as autonomous vehicles, wearable electronics, and implantable bioelectronics. Typically, the source (transmitter) and load (receiver) are coupled via a magnetic field, in which coils are used as energy couplers. Two objects with the same resonant frequency can exchange energy effectively, yielding a high WPT efficiency (*1*–*10*). The resonance maximizes the induced voltage across the rectifier at the load, improving the rectifier efficiency as well. Therefore, resonance has been applied widely in various types of wireless operation, such as in near-field WPT using coils (*2*, *11*, *12*) and cavities (*13*, *14*), including the industrial standards of Qi (*3*) and AirFuel (*4*), as well as midrange WPT using magnetic resonances (*1*, *6*).

In WPT, the quality factor (*15*) of the components must be maximized to achieve high efficiency (*1*, *6*). However, a high-quality factor inherently makes the WPT system have a narrow bandwidth and, hence, be vulnerable against external perturbations. Even a small change in the resonant frequency leads to a significant degradation in the WPT performance. The resonant frequency often varies for several reasons, including spatial displacement or orientation changes of the coils and variation in the load impedance (*1*, *6*–*9*, *16*). For WPT with a single source coil, several techniques have been proposed to maintain high efficiency despite such changes. For example, the operating frequency (*7*, *17*) or the components of the matching circuit (*16*, *18*) can be adjusted to keep the system at resonance. As another approach, extra coils can be added for the spacing between coils to be controlled, which scales the reflective impedance and maintains the system at resonance (*1*, *6*). However, such tuning methods require tracking the operation conditions, which is strenuous and, thus, limits the achievement of the seamless and efficient WPT.

Recently, a WPT mechanism that eliminates the need for active tuning has been proposed. It used a pair of transceivers (dimer) in parity-time (PT) symmetry to ensure WPT operation at resonant frequencies, which appear as real eigenvalues of the Hamiltonian operator describing the system dynamics (*8*, *9*, *13*, *19*, *20*). Note that while the Hamiltonian operator of a WPT system cannot be Hermitian because the system is not closed (e.g., energy goes in and out of the system), the reported systems, nevertheless, have the real eigenvalues. Specifically, if the Hamiltonian operator $\hat{H}$ of a system obeys PT symmetry $\left(\hat{H}\mathrm{PT}=\mathrm{PT}\hat{H}\right)$ and has a non-Hermiticity parameter under a certain threshold, it has entirely real eigenvalues even if the operator is non-Hermitian (*21*). This phase is referred to as unbroken PT symmetry, as opposed to broken PT symmetry in which the eigenvalues can be complex numbers even though $\hat{H}\mathrm{PT}=\mathrm{PT}\hat{H}$. Although the concept of PT symmetry originated from quantum physics (*21*), the similarity of the Schrodinger and electromagnetic wave equations broadened its applications into the fields of optics and electronics to unveil several exotic features, such as optical nonreciprocity (*22*, *23*), intrinsically single-mode lasers (*24*, *25*), unidirectional scattering (*26*, *27*), ultrasensitive sensors (*28*–*30*), and robust WPT (*8*, *9*, *13*, *19*, *20*).

**Fig. 1.**
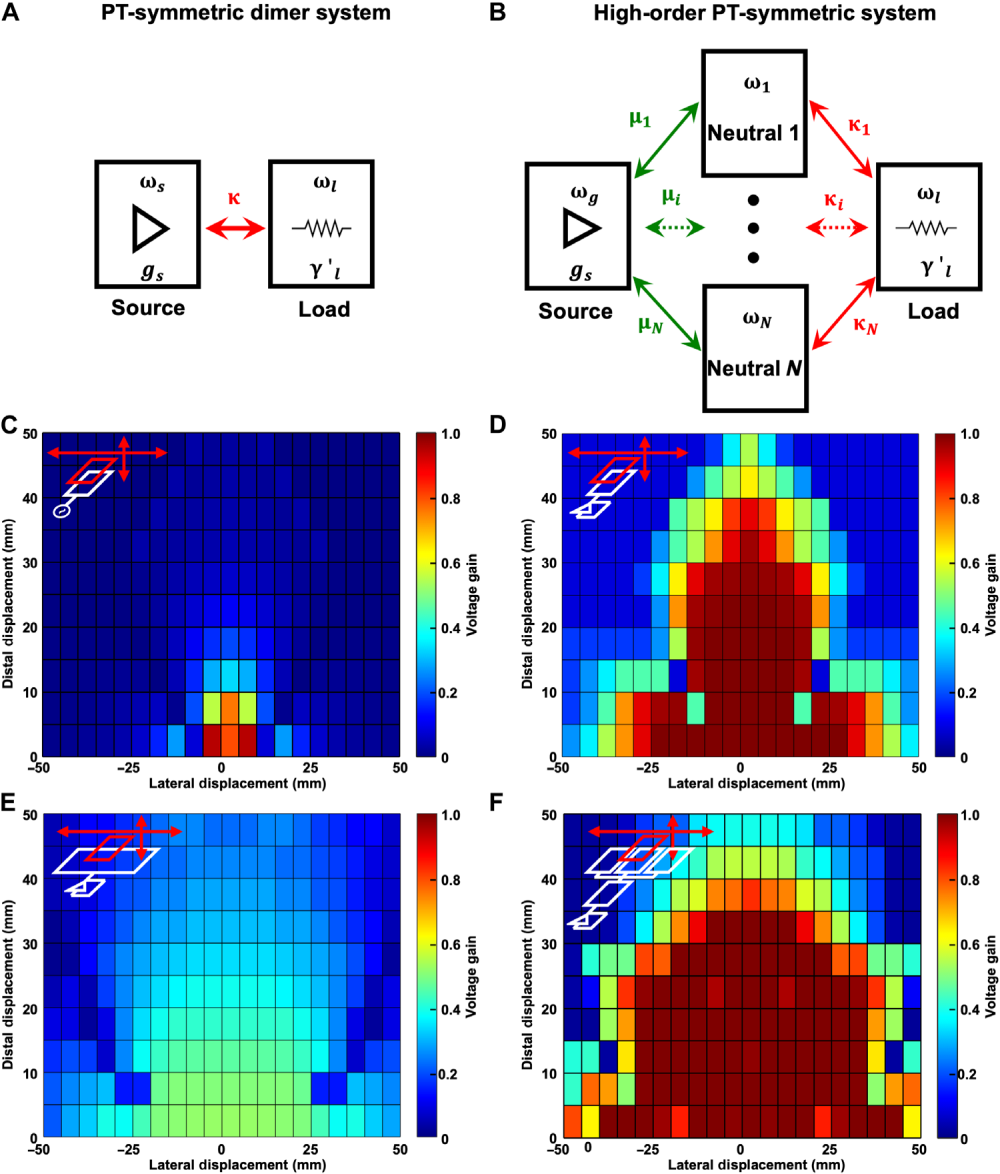
Schematics of the dimer and high-order PT-symmetric system and visualization of the enlarged operational area of the proposed high-order system. (**A**) Schematic of the PT-symmetric dimer system with the coupling rate κ, gain rate *g_s_* of the source (left), energy loss rate γ′*_l_* of the load (right), and their corresponding resonant frequencies ω*_s_* and ω*_l_*. (**B**) Schematic of the high-order PT-symmetric system with laterally aligned neutral elements. The *i*th neutral (*i* = 1, …, *N*) is coupled with the source and the load with the coupling rate μ*_i_* and κ*_i_*, respectively. The coupling rates μ*_i_* are adjusted to match κ*_i_* element-wise to achieve PT symmetry, i.e., μ*_i_* = κ*_i_*. For (A) and (B), all the coils in the resonators have the same dimension. (**C** to **F**) Simulated voltage gain (=|*V_l_*/*V_s_*|) in the operational area of (C) the conventional WPT system, (D) the PT-symmetric system with balanced coil sizes, (E) the PT-symmetric system with unbalanced coil sizes, and (F) the high-order PT-symmetric system (*N* = 3). The insets in the figures schematically represent the system setups. The dimension of all the coils are 30 mm by 18 mm, except the coil dimension for the source of (E) (30 mm by 60 mm), which is the same as the aperture of the three neutrals in (F). More details about the system setup are described in Materials and Methods.

The PT-symmetric WPT system (dimer system) is depicted schematically in Fig. 1A. The source and load resonators involve the coils of the same dimension and have the same resonant frequencies. As the transceiver pair is in the unbroken PT-symmetric phase, the saturable gain rate *g*_s_ at the source (left) compensates for the energy loss rate γ′_l_ at the load (right), reaching a steady state. This system self-tracks and oscillates at a variable resonant frequency to achieve a high power-transfer efficiency, because the voltage amplitudes are equally distributed between the source and load as long as they are strongly coupled with the coupling rate κ. Other reports (*31*, *32*) have deployed a neutral coil between the source and the load to enhance the operation range further in the distal direction by relaying the power. In the previous PT-symmetric systems, however, only a single coil interacts with the load coil. If the coupling between two coils drops below a certain threshold, the voltage gain (=|*V_l_*/*V_s_*|, where *V_s_* and *V_l_* are the voltage amplitudes of the source and load, respectively) and the efficiency degrade severely, limiting the coverage of the power transmission area and the tolerance of the misalignment between transceivers, especially in the lateral direction.

This motivates us to introduce the higher-order PT-symmetric system (Fig. 1B) using laterally aligned multiple neutral elements (center) arranged between the source (left) and load (right) for wide-range robust WPT. The coils in the resonators of the source and load as well as those in the neutrals have the same dimension. The *i*th neutral is energy-coupled to the load and source resonator with coupling rates of κ*_i_* and μ*_i_*, respectively. The system may become PT-symmetric by matching the coupling rates element-wise (μ*_i_* = κ*_i_*, *i* = 1, …, *N*, where *N* is the number of the neutrals). Unlike the conventional multiple-input–single-output (MISO) system, which requires auxiliary components such as a phase shifter, linear amplifier, or power inverters for each input coil (*33*–*35*), only the coupling capacitors between the source and neutral resonators are required and controlled to make the system PT symmetric. The elimination of the expensive auxiliary components makes the system highly cost-effective. Moreover, our approach decreases the design parameter dimension from *R* × *C^N^* (frequency × gain amplitudes and phases of *N* coils in the conventional MISO system) to *R*^*N+*1^ (*N +* 1 variable capacitances in the proposed system), significantly reducing the overall system complexity, which is essential for the seamless, dynamic operation of the WPT system.

The simulated voltage gains plotted in Fig. 1 (C to F) compare the operational areas of aforementioned WPT systems with the proposed high-order PT-symmetric system. Figure 1C shows the voltage gain obtained from the conventional WPT system in which the dimension of the source coil is balanced with that of the load coil (Fig. 1C, inset). Because the operating frequency is fixed regardless of the coupling between the source and load, the voltage gain significantly drops sensitively with the displacement of the load, resulting in a very narrow coverage area of the system as shown in Fig. 1C. On the other hand, Fig. 1D shows the voltage gain of the PT-symmetric dimer system with the balanced coils (Fig. 1D, inset). The automatic adjustment of the oscillation frequency with respect to the displacement allows a wide operational area along the distal direction, but not along the lateral direction. To broaden the area of the WPT in the lateral direction, an enlarged source coil can be considered (Fig. 1E, inset), which insists the single-input–single-output (SISO) system. Although the dimension of the source is unbalanced with the load, the system can remain PT symmetric by matching the resonant frequencies of transceivers (*28*) through the adjustment of the resonating capacitors. Regardless of the PT symmetry, however, the overall voltage gain is quite low because of the unbalanced inductances between the source and the load. This is analogous to the non-unity voltage ratio appearing at a transformer with a different turn ratio. Moreover, because the coefficient of coupling *k* between unbalanced coils is small, the voltage gain begins to degrade at a shorter distance, limiting the coverage area in the distal direction, as seen from the normalized voltage gain in fig. S1.

Compared to those systems, the proposed system shows the largest operational area (Fig. 1F). Specifically, this example includes a total of five units (Fig. 1F, inset), including a source, a load, and three neutral units, to form a pentamer system. By maintaining the PT symmetry among the five units, the voltage gain of the pentamer system can be constantly high over the largest area. Such uniform voltage gain is essential to ensure robust WPT operation and to protect the voltage-sensitive interfaces of small-power devices, such as bioelectronic devices. The effectiveness of the proposed WPT system was proven by both theoretical calculations and experimental demonstrations. The potential of the flexible WPT system was also demonstrated, with the objective of enabling various mobile and bio-integrated electronic applications (*36*, *37*).

## RESULTS

### Theoretical analysis of the PT-symmetric oligomer system

**Fig. 2.**
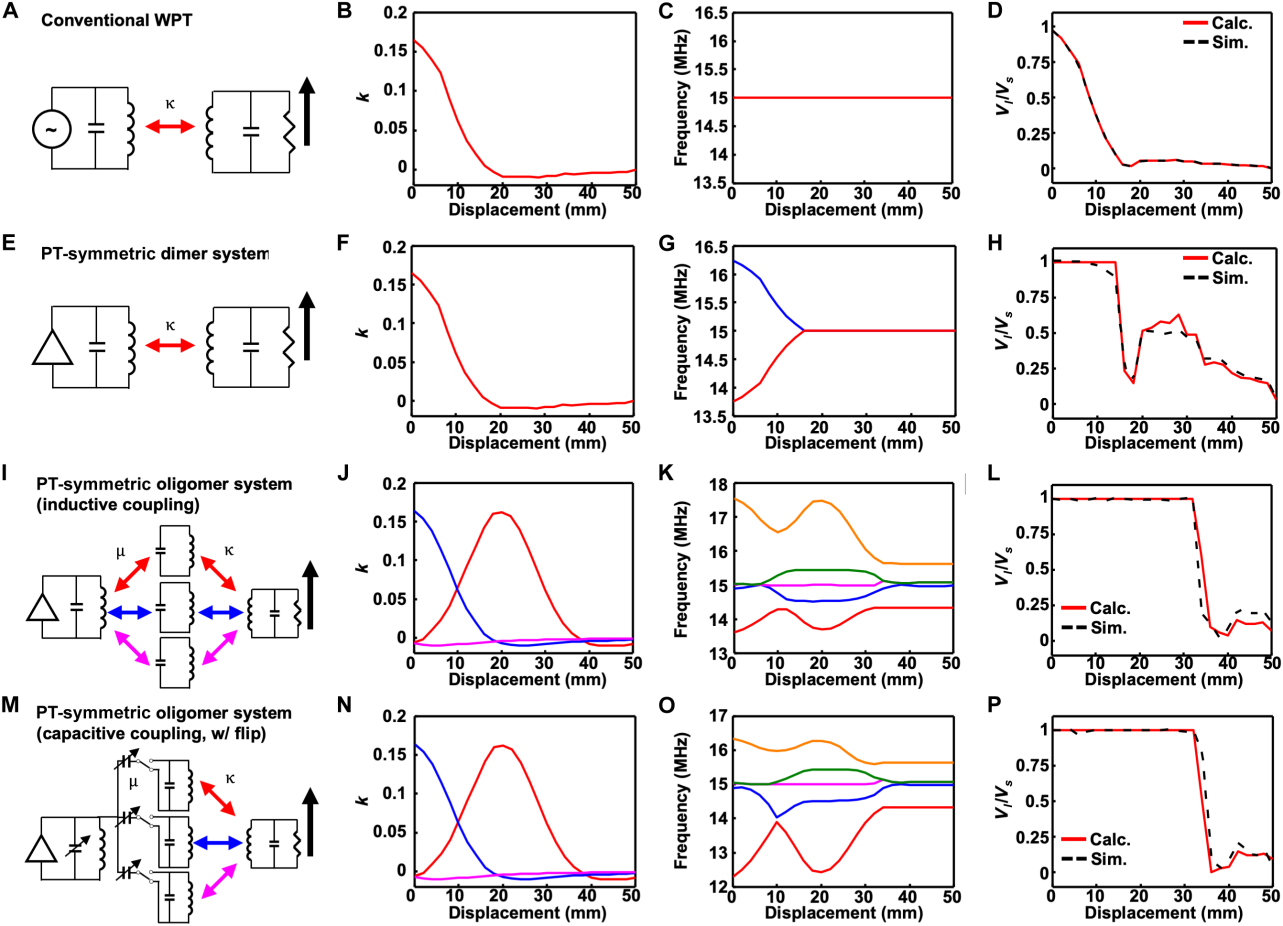
Schematic diagrams and the coupling coefficient *k*, operation frequency, and voltage gain relative to the lateral displacement between the source and load of four different RF WPT configurations in a developmental sequence. The coverage of both calculated and simulated voltage gain is enhanced, and complexity is reduced as the system develops from the top to bottom row. (**A** to **D**) Conventional inductively coupled WPT system. (**E** to **H**) PT-symmetric dimer system. (**I** to **L**) PT-symmetric oligomer system with a homogeneously coupled configuration. (**M** to **P**) PT-symmetric oligomer system with a heterogeneously coupled configuration. The red, blue, and pink lines in (J) and (N) correspond to the coupling between the load and neutral unit at the top, center, and bottom, respectively, whereas the load is laterally displaced along the black arrows in (I) and (M). The lines in (K) and (O) show the eigenvalues (also called eigenfrequencies) according to the lateral displacement.

Figure 2 depicts the theoretical characteristics of four different RF WPT configurations in a developmental sequence. For each system, a schematic diagram is provided, and the coefficient of coupling *k*, operation frequency, and voltage gain are presented according to the lateral displacement between the source and load. These systems are analyzed with both analytical calculation and computer simulation (Materials and Methods).

Figure 2A provides a schematic diagram of a conventional WPT system. Normally, a WPT system necessitates impedance matching to establish resonance between transceivers at a specific transfer distance and alignment. When the lateral displacement between the transceivers varies as indicated by the black arrow, *k* between the transceivers changes (Fig. 2B) and alters the resonant frequency as well. As the operating frequency does not change with the system variations (Fig. 2C), the voltage gain (=|*V_l_*/*V_s_*|) of the conventional WPT system quickly decreases with the displacement (Fig. 2D). Unless the coupling between the transceivers is actively monitored to tune the operating frequency under every set of conditions, this outcome is unavoidable. Therefore, the conventional WPT system exhibits obvious drawbacks under the displacements.

To overcome the susceptibility to operational condition changes, a PT-symmetric dimer system was proposed (Fig. 2E) (*8*, *9*, *20*). According to the coupled-mode theory (*38*), the system dynamics for the coupled resonators are represented by

$$\frac{d}{\mathrm{dt}}[\begin{array}{c} V_{s}\\ V_{l} \end{array}]=[\begin{array}{cc} i\omega_{s}+g_{s} & -i\kappa \\ -i\kappa & {i\omega }_{l}-{\gamma \prime }_{l} \end{array}][\begin{array}{c} V_{s}\\ V_{l} \end{array}]=\frac{H}{i}[\begin{array}{c} V_{s}\\ V_{l} \end{array}]$$ (1)

where *H* is a Hamiltonian matrix describing the system, and ω*_s_* (or ω*_l_*) refers to the self-resonant frequency of the source (or load) resonator. For PT-symmetric operation, the resonant frequencies of the source and load are set to be equal as ω_0_ := ω*_s_* = ω*_l_*. The overall gain of the source resonator is *g_s_* = *g*_*s*0_ − γ_*s*0_, where *g*_*s*0_ is the gain rate and γ_*s*0_ is the intrinsic loss rate of the source resonator. Likewise, γ′*_l_* = γ*_l_* + γ_*l*0_ is the overall loss rate of the load resonator, where γ*_l_* is the output loss rate and γ_*l*0_ is the intrinsic loss rate due to thermal and radiation losses. In addition, κ (= ω_0_*k*/2) is the coupling rate between the source and load resonator.

In the unbroken phase of PT symmetry where the coupling rate κ exceeds the loss rate $\gamma_{l}^{\prime }$, the nonlinear amplifier in the source provides energy with saturated gain rate *g_s_* that exactly balances the loss $\gamma_{l}^{\prime }$ in the load ($g_{s}=\gamma_{l}^{\prime }$). Because the voltage amplitude of the load equals that of the source in the unbroken phase of PT symmetry (*8*, *9*), a high efficiency can be achieved. This feature makes the dimer system robust especially when the transceiver coils are axially aligned. The coupling coefficient between axially aligned coils decreases rather slowly with increasing distance. Nevertheless, the system is vulnerable to the lateral displacement between the transceivers as indicated by a black arrow (Fig. 2E). The coupling coefficient decays rapidly as the displacement increases (Fig. 2F). In the unbroken phase, the system has two different real eigenfrequencies $\omega =\omega_{0}\pm \sqrt{\kappa^{2}-\gamma \prime_{l}^{2}}$ (Fig. 2G), and the operating frequency is selected to be one of them to maintain the highest power transfer efficiency. By self-adjusting the operational frequency, the voltage gain is kept high up to ~10-mm displacement but begins to decrease when the system becomes PT asymmetric $(\hat{H}\mathrm{PT}\neq \mathrm{PT}\hat{H}),$ where the gain is not balanced with the loss ($g_{s}\neq \gamma_{l}^{\prime }$) and the eigenfrequencies degenerate (Fig. 2H) (*8*, *9*, *13*, *19*, *20*, *39*). Hence, although the dimer system is effective because of self-adjustment tuning, there is a significant need for further improvement to achieve robust WPT under lateral displacements.

Therefore, we propose an oligomer system that includes multiple neutral units to overcome the vulnerability against lateral displacements. Although the neutrals and load are inductively coupled using the coils as usual, the coupling between the neutrals and source can be made either homogeneous (inductive) or heterogeneous (capacitive). When a neutral layer is added, the system dynamics is described by

$$\frac{d}{\mathrm{dt}}[\begin{array}{c} V_{s}\\ V_{1}\\ V_{2}\\ \vdots \\ V_{N}\\ V_{l} \end{array}]=\frac{H}{i}[\begin{array}{c} V_{s}\\ V_{1}\\ V_{2}\\ \vdots \\ V_{N}\\ V_{l} \end{array}]$$ (2)

where the Hamiltonian *H* can be expressed in general as

$$H=[\begin{array}{cccccc} -(\omega_{s}-\Delta \omega_{s})+{\mathrm{ig}}_{s} & -\mu_{1} & -\mu_{2} & \cdots & -\mu_{N} & 0\\ -\mu_{1} & -(\omega_{1}-\Delta \omega_{1}) & -\kappa_{12} & \cdots & -\kappa_{1N} & -\kappa_{1}\\ -\mu_{2} & -\kappa_{12} & -(\omega_{2}-\Delta \omega_{2}) & \cdots & -\kappa_{2N} & -\kappa_{2}\\ \vdots & \vdots & \vdots & \ddots & \vdots & \vdots \\ -\mu_{N} & -\kappa_{1N} & -\kappa_{2N} & \cdots & -(\omega_{N}-\Delta \omega_{N}) & -\kappa_{N}\\ 0 & -\kappa_{1} & -\kappa_{2} & \cdots & -\kappa_{N} & -(\omega_{l}-\Delta \omega_{l})-i\gamma_{l}^{\prime } \end{array}]$$ (3)

Here, *V_m_*(*m* = *s*, *l*, 1, 2, …, *N*) refers to voltage amplitudes of the resonators. The subscripts *s* and *l* indicate the source and load, respectively, and the numerical subscript *i* (*i* = 1, 2, …, *N*) represents the *i*th neutral unit. Likewise, ω*_m_*(*m* = *s*, *l*, 1, 2, …, *N*) denotes the self-resonant frequency of each resonator, and Δω*_m_* is a frequency offset that may appear in the coupled-mode theory model due to the coupling between resonators. κ*_ij_* (*i* ≠ *j*) indicates the coupling rate between the *i*th and *j*th neutral units. We assume that the source and load are not directly coupled (*H*_1, *N* + 2_ = *H*_*N* + 2,1_ = 0). The representations of *H* including the frequency offset in terms of the circuit components are given in text S1 for each coupling configuration (each circuit diagram is shown in fig. S2).

Similar to the dimer case, we made the oligomer system with the neutrals to be PT symmetric ($\hat{H}\mathrm{PT}=\mathrm{PT}\hat{H})$; it guarantees the equal amplitude distribution between the source and the load, yielding a high efficiency. For this purpose, we first control μ*_i_* to be the same as κ*_i_* element-wise (the full conditions of the PT symmetry are described in text S2). Intuitively, it should be beneficial to excite the neutral with a strong coupling κ*_i_* to the load via a strong coupling μ*_i_* from the source. For example, when the number of the neutrals *N* is three, as the load is laterally displaced along the direction of the black arrow (Fig. 2I), the distances between the first/second neutral resonator (neural unit at the bottom/center) and the load increase, and thus the coupling rates κ_1_ and κ_2_ decrease (Fig. 2J, blue/pink lines). On the other hand, as the load approaches the third neutral resonator (neural unit at the top), the coupling rate κ_3_ between the top and load resonator increases (Fig. 2J, red line). According to this displacement, we adjust the couplings from the source by increasing μ_3_ and decreasing μ_1_ and μ_2_, so that the overall system may become PT symmetric.

In addition to matching the coupling strengths, the gain *g_s_* should exactly compensate for the loss $\gamma_{l}^{\prime }$ of the WPT system to be PT symmetric; *g_s_* = $\gamma_{l}^{\prime }$ (text S2). The gain element of the WPT system was implemented by a nonlinear amplifier (fig. S3). The nonlinear dynamics in the saturation of the gain ensures that the WPT system should reach a steady state of oscillation instead of amplitudes diverging to infinity. The real eigenvalues of the Hamiltonian can potentially be the oscillating frequency of the WPT system at the steady state. Specifically, among numerous steady-state modes with the corresponding real eigenvalues for a variable gain *g_s_*, the mode requiring the lowest gain *g_s_* is selected as the operating mode (*8*). Following the method presented in text S3, fig. S4A shows that the lowest (saturated) gain with the real eigenvalues is equal to $\gamma_{l}^{\prime }$ up to a displacement of ~30 mm, which makes the system PT symmetric by satisfying the condition $g_{s}=\gamma_{l}^{\prime }$. Within this range, the amplitude of the load is equal to the source, providing the voltage gain of unity (Fig. 2L). Besides, the real eigenvalues corresponding to the saturated gain *g*_*s*, sat_ are shown in Fig. 2K. The system self-tracks the oscillation frequency as one of the real eigenvalues, so that the voltage gain and efficiency are maintained high regardless of the displacement. Beyond the range of ~30 mm, in contrast, the system is not PT symmetric anymore because the saturated gain is less than $\gamma_{l}^{\prime }$ (fig. S4A) and the voltage gain begins to deteriorate.

Although the inductively coupled system between the source and neutral layer shows positive outputs, it could be further elaborated to become more practical. To obtain the response as designed, the coupling between the source and neutral layer should be controlled as the coupling between the neutral layer and load varies. However, to control the level of inductive coupling, the relative distance or orientation between the coils should be changed, which requires additional moving parts that are mechanically controllable and makes the overall system bulky and complicated.

On the other hand, the controllable capacitive coupling can be easily implemented with a variable capacitor connecting the source and neutrals, which is more favorable for consumer and biomedical electronics because mechanically moving parts are not required. To satisfy the PT-symmetric condition, one may attempt to set the coupling rate $\mu_{i}=\omega C_{\mathrm{si}}/(2\sqrt{C_{s}C_{i}})$ to be equal to |$\kappa_{i}|=\omega |M_{\mathrm{il}}|/(2\sqrt{L_{i}L_{l}})$. Nonetheless, simple alteration of the coupling type can rapidly decrease the efficiency. At a certain distance, the mutual inductance *M_il_* changes the sign to yield μ*_i_* = −κ*_i_*, rather than μ*_i_* = κ*_i_*, which breaks the PT symmetry and can result in a decrease in the voltage gain.

This issue can be solved by allowing the flip of each neutral coil (Fig. 2M), which effectively reverses the sign of κ*_i_*. Figure S3 provides a detailed circuit diagram. When the coefficient of coupling changes in the same manner as in the previous inductively coupled configuration (Fig. 2N), such modifications may restore the PT symmetry (μ*_i_* = κ*_i_*), resulting in a similar trend in the split of the eigenvalues (Fig. 2O) and a uniformly high voltage gain over the displacement range up to ~30 mm (Fig. 2P). As in the inductive coupling configuration, the system is PT symmetric ($g_{s,\text{sat}}=\gamma_{l}^{\prime }$) within the range up to ~30 mm and PT asymmetric ($g_{s,\text{sat}}<\gamma_{l}^{\prime }$) beyond this range (fig. S4B). When the simulations were repeated for various positions of the load over the two-dimensional plane, the PT-symmetric oligomer system provided robustness against misalignment as well as the variable distance between the source and load, as shown in the voltage gain (Fig. 1F).

### Experimental validation of the PT-symmetric pentamer system with capacitive coupling

**Fig. 3.**
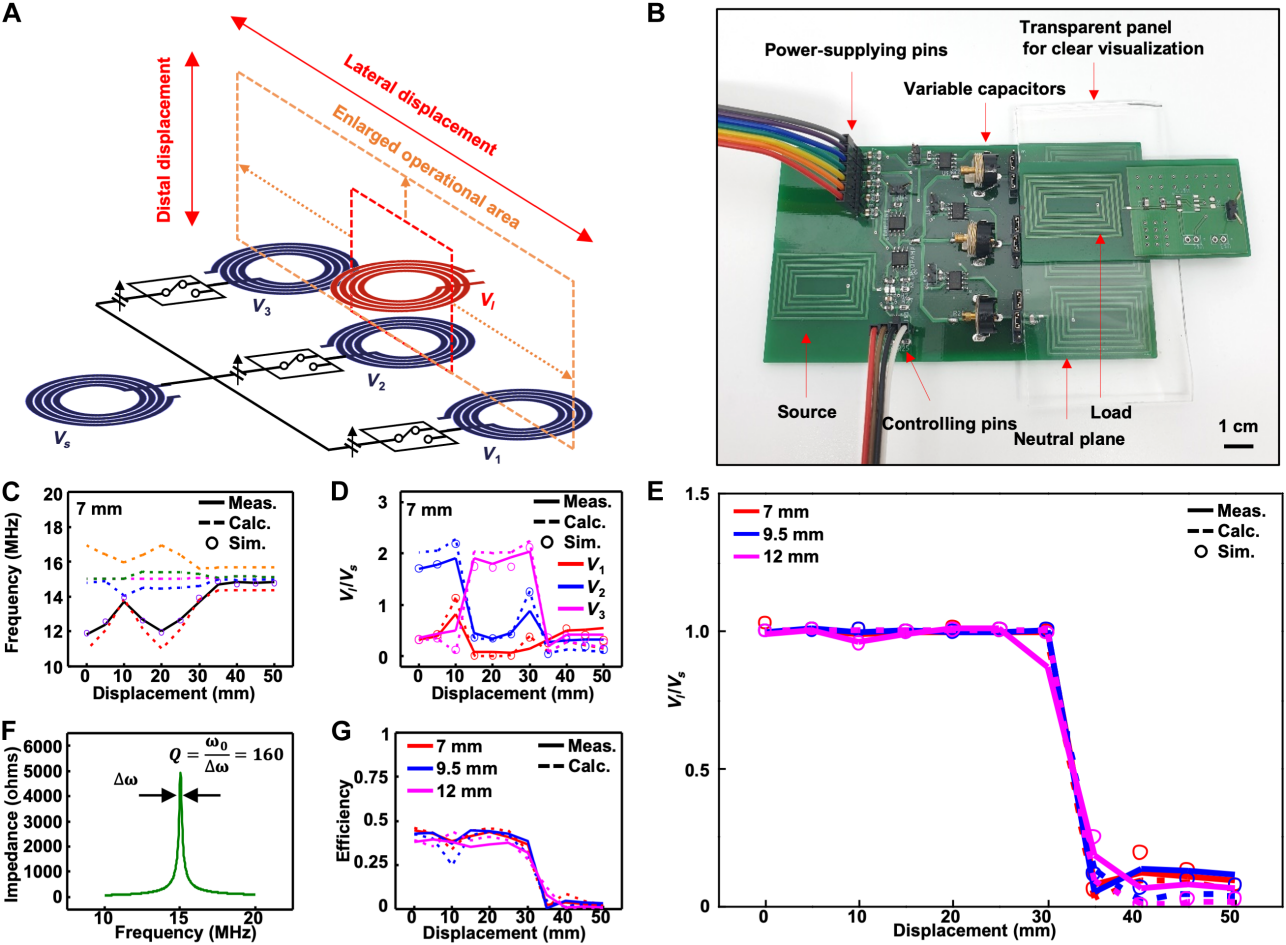
Experimental demonstration of heterogeneously coupled and flippable neutral units in the PT-symmetric pentamer system. (**A**) Schematic diagram of the proposed system. The operational area is enlarged with distal and lateral displacement. (**B**) Prototype of the proposed system is made on a commercial FR4 printed circuit board. The responses of the system such as eigenvalues, voltage amplitudes, and voltage gains are displayed with the lateral displacement of the load. (**C**) Multiple eigenvalues versus lateral displacement in the pentamer system. One of them was chosen as the operating frequency of the system and is indicated by the black solid line. (**D**) Measured voltage amplitudes of the three neutrals arranged between the source and load. The results resemble the calculated and simulated data fairly well. (**E**) Constant voltage gain acquired from the system with distal displacements of 7, 9.5, and 12 mm. The measurements follow the calculated and simulated results fairly well. (**F**) Impedance profile of a coil from which the quality factor can be deduced (~160). (**G**) Constant efficiency acquired from the system with three different distal displacements. The measurements follow the calculated and simulated results fairly well.

Using the heterogeneously coupled and flippable neutral units in the PT-symmetric oligomer system enables a wide range of robust WPT under both lateral and distal displacements as illustrated in Fig. 3A. On the basis of the circuit simulation, we constructed a prototype device (Fig. 3B and Materials and Methods). The self-resonant frequency of five resonators was set to be near ω_0_ = 2π × 15 (Mrad/s). A transparent panel was inserted between the transmitter and receiver units in Fig. 3B to visually distinguish two units. In the actual experimental setup (fig. S5A), the panel was removed and the load resonator was placed midair using a custom-made plastic holder (fig. S5B) that could fix the distal and lateral displacements between the neutral plane and the load resonator.

The system response was measured as the load resonator was moving along the lateral direction, as described in Fig. 3A. For each displacement of the load, the coupling rates μ*_i_*, the flipping elements, and the source capacitor are manually adjusted to fulfill the PT-symmetric conditions (Materials and Methods). Figure 3 (C to G) shows the response of the system when the distal distance between the neutral plane and load resonator was 7 mm and the lateral displacement was varied between 0 and 50 mm. Figure 3C demonstrates that the system selects one of five eigenvalues as the operating frequency. The vector of voltage amplitudes (***V*** = [*V_s_*, *V*_1_, *V*_2_, *V*_3_, *V_l_*]*^t^*) is the eigenstate of the Hamiltonian corresponding to the specific eigenvalue. The simulated and measured voltage amplitudes normalized with the source voltage *V_s_* well match the calculated eigenstates in Fig. 3 (D and E). As the load moves toward the third neutral coil and away from the first and second ones, the coupling rate μ_3_ increases to match κ_3_, while the coupling rates μ_1_ and μ_2_ are reduced. Thus, the voltage amplitude of the third neutral coil (*V*_3_) increases, but the amplitudes of the other neutrals decrease. This helps to reduce the energy loss in the neutrals weakly coupled to the load. When the coupling rates are stronger than a certain threshold (*x* = 0 to 30 mm), the experimental results confirm that the system is PT symmetric and that there exists a simultaneous eigenstate ***V*** for the *PT* and $\hat{H}$ operators, yielding a nearly constant voltage gain of unity (Fig. 3E).

Although the energy loss by the neutral elements was ignored (γ_*i*0_ = 0 for *i* = 1, 2, …, *N*), for the formalism of PT symmetry in Eq. 3, this loss can be restored to compute the (power-transfer) efficiency once the voltage amplitudes of the resonators are determined. The efficiency in the steady state is given by

$$\eta =\frac{\gamma_{l}C_{l}{|V_{l}|}^{2}}{\gamma_{s0}C_{s}{|V_{s}|}^{2}+\sum_{i=1}^{N}\gamma_{i0}C_{i}{|V_{i}|}^{2}+{\gamma \prime }_{l}C_{l}{|V_{l}|}^{2}}$$ (4)

where γ_*i*0_ refers to the intrinsic loss rate due to thermal and radiation losses of the *i*th neutral resonator. The intrinsic loss rate of the *m*th resonator (γ_*m*0_ = ω_*m*_/2*Q*,*m* = *s*,*l*,1,2,…,*N*) can be deduced from the quality factor *Q*. According to the bandwidth of the measured impedance profile (Fig. 3F), the quality factor of the resonators made of a rigid conductor can be obtained as ~160. The efficiency obtained from Eq. 4 is robustly maintained throughout the strongly coupled region (0 to 30 mm), as shown in Fig. 3G. The relatively low efficiency compared to the near-unity voltage gain is mainly due to the energy losses γ_*i*0_ of the multiple neutrals in Eq. 4. In addition, while the previous works (*8*, *9*) used a large coil size to harness a high unloaded quality factor above 300, we chose to fabricate the coil (*Q* ~ 160) on a printed circuit board to reduce the system size and the cost. Higher efficiencies can be achieved by increasing the thickness of the patterned coil, which effectively increases the quality factor. Figure S6 shows the simulated efficiency of the system when the thickness of the patterned coil increases from 30 to 70, 150, or 400 μm, while the distal distance is fixed as 7 mm and the load impedance is 1 kilohm. It shows that the overall efficiency can be increased by increasing the thickness of the patterned coil. As the load goes far beyond the range of the neutral coils (>30 mm), the system is not PT symmetric ($\hat{H}\mathrm{PT}\neq \mathrm{PT}\hat{H}$), and the overall efficiency decreases significantly.

Figure 3 (E and G) and fig. S7 also show the system response when the distal distance between the neutral and receiver resonator is shifted from 7 to 9.5 or 12 mm. From these results, it is observed that the overall results are similar to those when the distance is 7 mm. Such data prove that the voltage gain and efficiency remain high against lateral and distal displacements.

### Application of flexible receivers to the capacitively coupled PT-symmetric pentamer system

Implantable bioelectronic devices have been widely used to monitor physiological and electrophysiological signals (e.g., heart signal, glucose, and blood pressure) and/or to stimulate organs and nerves (e.g., heart and auditory nerves) (*40*–*42*). However, such devices mostly rely on batteries that require periodic surgery for replacement (*42*–*44*). As devices including batteries are rigid, mechanical mismatch between the device and soft human body occurs, which imposes continuous stress on the target tissue and causes inflammatory responses. The conventional RF-based WPT can be applied to eliminate or at least minimize such issues (*45*–*47*). However, misalignments between transceivers caused by dynamic motions and the soft and complex form of the human body often hinder reliable power supply. The PT-symmetric oligomer WPT system with flexible receivers could be a solution. A WPT system whose voltage gain is robustly maintained over an extended range can provide reliable power supply even in flexural and dynamic human body parts.

**Fig. 4.**
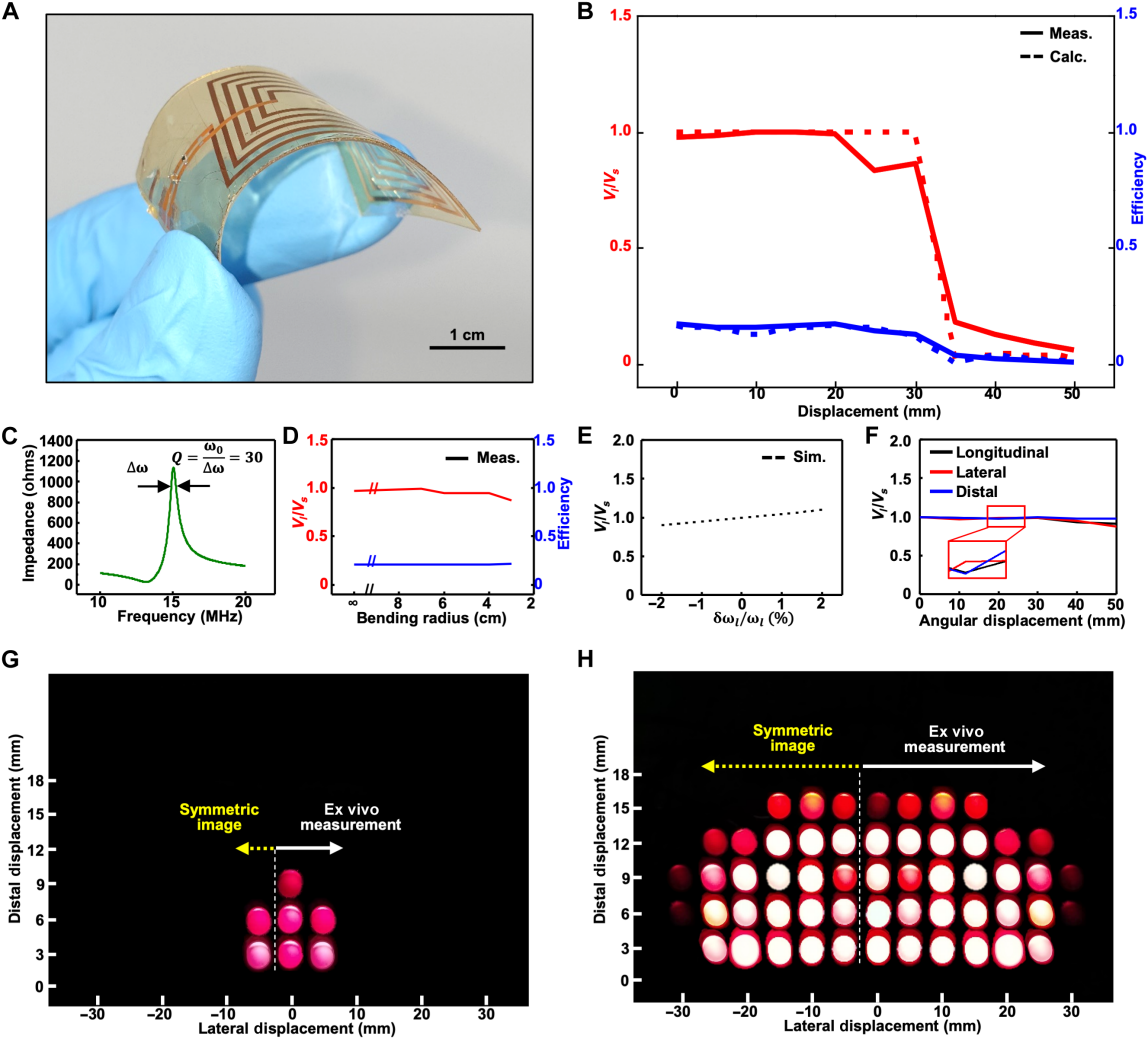
Characterization and ex vivo demonstration of the PT-symmetric pentamer system using a flexible load resonator. (**A**) Photograph of the flexible load resonator. The design of the coil is identical to the rigid coil, but the flexible coil was fabricated in the academic microfabrication processes. (**B**) Constant voltage gain acquired from the system at different lateral displacements from 0 to 50 mm in 5-mm increments. (**C**) Bandwidth of the measured impedance profile of a flexible coil, from which the quality factor can be deduced (~30). (**D**) Constant voltage gain and efficiency of the flexible load resonator acquired from the pentamer system at different bending radii. (**E**) Simulated voltage gain in the pentamer system as a function of the self-resonant frequency change of the flexible load resonator. (**F**) Measured voltage gain in the pentamer system as a function of the angular displacement of 0°, 10°, 20°, 30°, 40°, and 50° about longitudinal, lateral, and distal direction. (**G**) Images for the ex vivo demonstration using porcine tissue in the conventional WPT system. (**H**) Images for the ex vivo demonstration using porcine tissue in the proposed heterogeneously coupled PT-symmetric pentamer system. A light-emitting diode (LED) is used in the resonator circuit to demonstrate the reliable WPT to the load resonator through the tissue under distal and lateral displacements. The images of the lighted LEDs at various distal and lateral displacements (shown in fig. S13) are integrated into an image for effective visualization of the WPT coverage and comparison of each WPT system.

The robustness of the WPT system was tested using a flexible load resonator. Figure 4A depicts the flexible coil used for the experiment. Combined with a discrete component capacitor, the coil forms a flexible resonator. The thin film substrate enables the resonator to be flexed. The coil was fabricated with copper on a silicon wafer and was transferred to an elastomeric substrate. Other emerging conductive materials, such as graphene, which features high conductivity and excellent mechanical properties, are promising candidates for the wireless powering, especially for nanoscale powering device applications (*48*–*51*). However, there are remaining challenges for their widespread applications, such as further conductance enhancement. Figure S8A describes the fabrication steps in detail, and fig. S8B is the exploded view for clearer indication of each layer.

The response of the flexible load resonator was measured throughout the distal and lateral displacements. Figure S9 confirms that the flexible load resonator exhibits similar profiles to those of the conventional printed circuit boards under the same experimental conditions. In particular, the voltage gain (fig. S9, C, G, and K) of the flexible resonator (~1.0) is nearly the same as that of the rigid resonator (~1.0). However, the efficiency (fig. S9, D, H, and L) of the flexible resonator (~0.18) is decreased compared to that of the rigid resonator (~0.4). The representative voltage gain and efficiency of the flexible load resonator are shown in Fig. 4B for the case of a distal distance of 7 mm. The efficiency is lowered because the DC resistance increases from ~0.3 ohms of the rigid coil to ~1.8 ohms of the fabricated flexible coil. The high resistance of the flexible coil reduces the quality factor to ~30, as seen from the impedance profile in Fig. 4C, which degrades the efficiency. Nonetheless, the similarities between the rigid and flexible systems with distal displacements of 7, 9.5, and 12 mm prove that the proposed system is highly robust against lateral and distal displacements even with the unconventional flexible load.

The robustness of the system was investigated under bent conditions (fig. S10, A to G), resembling the deformation with the human body. Figure 4D shows the measured system response when the flexible load resonator undergoes bending. The voltage gain (~1.0) and efficiency (~0.18) of the WPT system are fairly stable up to a bending radius of 3 cm (Fig. 4D), which is considered sufficient for covering most human organs (*52*, *53*). The experimental results also show that the change in the operating frequency was negligible and that the voltage amplitudes of the neutrals remained unchanged (fig. S11, A and C). The robustness against flexural deformation can be again explained by the coupled-mode theory. The bending of the load coil affects the inductance of the load resonator and changes the self-resonant frequency of the load (ω_*l*_). For example, the bending radius of 3 cm shifts the resonant frequency (δω_*l*_/ω_*l*_) by about 1%. Because the resonant frequency change of the coil is subtle, it does not markedly affect the real eigenvalue and corresponding eigenstates (*V*_*i*_/*V*_*s*_) and voltage gain (*V*_*l*_/*V*_*s*_) of Eq. 3, as observed from the simulation results of fig. S11 (B and D) and Fig. 4E, respectively. They all exhibit negligible variations in the range of ∣δω_*l*_/ω_*l*_∣ ≤ 2%.

Figure 4F and fig. S12 (A to F) show the experiments to characterize the system response when the flexible load resonator rotates in the longitudinal (fig. S10, H to N), lateral (fig. S10, O to U), and distal (fig. S10, V to AB) direction. For the rotations up to 50°, which cover a reasonable range of the rotations in practice, the voltage gain of the WPT system (~1.0) is fairly stable (Fig. 4F). The experimental results also show that the change in the operating frequency is negligible and that the voltage amplitudes of the neutrals remained unchanged (fig. S12, A, C, and E and fig. S12, B, D, and F, respectively). Such robust system responses to the rotations are because the angular rotation does not significantly affect the Hamiltonian of Eq. 3. For example, the coupling coefficient between the middle neutral and the load resonator is shifted from 0.164 at the rotation of 0° to 0.154 at the rotation of 50° in the distal direction. The adjustment of μ*_i_* per the change of κ*_i_* can preserve the PT symmetry of the Hamiltonian, achieving the voltage gain of near unity for the rotation range under consideration.

The potential for bio-application and the robustness of the proposed system were verified on porcine tissue ex vivo and compared to the conventional WPT system. The flexible load resonator was attached to the complexed surface form of soft porcine tissue (thickness of 3 mm), and a light-emitting diode (LED) was integrated to the resonator for easy visualization of WPT performance (fig. S13, A and B). The circuit of the flexible load resonator was modified to convert AC voltage to DC voltage to power the LED (fig. S13C). The system was tested with the load at various displacements for the comparison of the WPT using the PT-symmetric oligomer system (fig. S13D) and the conventional system (fig. S13E). The lighted LED images were integrated into one image for each system to compare the WPT range and verified that the proposed system exhibits continuous and reliable WPT throughout the broader distal and lateral displacements than the conventional system (Fig. 4, G and H).

## DISCUSSION

We reported a robust WPT system using heterogeneously coupled and flippable neutrals with PT symmetry, supported by background theory and experimental demonstrations. The proposed PT-symmetric oligomer system enlarges the operational area by using laterally aligned neutral elements. Although it requires additional components to implement multiple neutral elements, the system is considerably simpler than the conventional MISO WPT system because the added elements can be easily managed by variable capacitors without necessitating auxiliary components, such as phase shifters and linear amplifiers, for each element. Moreover, the nonlinear circuitry at the gain element establishes the PT symmetry so that the system itself can track the varying resonant frequency per change in the operational conditions. Consequently, the system becomes insensitive to changes in the operational conditions and provides a wide operational area compared to the conventional inductively coupled RF WPT system, enabling RF WPT in more diverse applications. For example, the WPT system should be effective for biomedical devices because it can reliably endure various deformations and uncertainties in in vivo environments. To adapt to such operational conditions further, the mechanical property was modified by inducing flexibility in the load resonator. For verification, measurements, simulations, and ex vivo demonstrations under flexural deformations were conducted and showed the potential usability of this system.

While the proposed WPT system self-selects the operating frequency that maximizes the voltage gain and reduces the dimension of design parameters from *R* × *C^N^* to *R*^*N+*1^ for *N* neutrals, the coupling capacitors are still needed to be actively tuned for the system to be PT symmetric. Such a tuning process can be automatized by replacing the manually tunable capacitors between the source and the neutrals with electronically tunable ones. This also helps to miniaturize the system size, which is favorable for the wearable device applications (fig. S14). The automatic tuning process can be implemented by monitoring either the received power at the receiver through a separate communication channel (*3*, *4*) or the input impedance at the source side (*33*), which needs further development in the future. Furthermore, the gain element implemented with an operational-amplifier circuit can be improved by using a power-efficient switch-mode amplifier to reduce the inherent loss and enhance the overall system efficiency (*9*). The compatibility of the switch-mode amplifier and the neutral elements needs to be investigated in the future. Last, further studies are necessary to enhance the efficiency and biocompatibility of the implantable bioelectronic devices by reducing the coil resistance and fabricating the coil to be stretchable (*45*, *46*), respectively.

## MATERIALS AND METHODS

### System setup for Figs. 1 and 2

The systems to obtain the voltage gains shown in Fig. 1 (C, D, and F) are identical to the systems in Fig. 2 (A, E, and M, respectively). The inductor of all the resonant tanks in Fig. 2 was a five-turn rectangular coil with a line thickness, line width, line spacing, and size of 30 μm, 0.7 mm, 0.8 mm, and 30 mm by 18 mm, respectively, and an inductance of 520 nH. The enlarged source coil of Fig. 1E differs only in the area as 30 mm by 60 mm. For the simulation with the neutrals in Fig. 2 (I and M), three neutral coils with the same coil dimensions were included, where the center-to-center distance between the coils was 20 mm.

For each displacement, the coupling between the coils was simulated using a commercial electromagnetic simulator (High Frequency Simulation Software, Ansys). The coupling coefficients *k* were exported to a commercial circuit simulator (Advanced Design System, Keysight Technologies). In the circuit simulator, the resonators nominally used a fixed capacitance of 216.5 pF to have a self-resonant frequency of 15 MHz, except that the source resonator in Figs. 1F and 2M used a variable capacitance to achieve PT symmetry for each displacement (see texts S1 and S2). The gain elements were implemented with the noninverting amplifier configured by an LM6171 operational amplifier and (*R*_1_, *R*_2_) = (5, 10) kilohms with a feedback resistance *R*_f_ of 68 ohms. Figure S3 provides full circuit schematics including the voltage followers to read the voltage amplitude with minimal perturbations and switches to enable coil flipping. The rightmost column of Fig. 2 shows the voltage gain (=∣*V_l_*/*V_s_*∣) obtained from the calculations and the circuit simulations when the load resistor *R_l_* of 4.5 kilohms was connected at the receivers.

### Electronic components to build the pentamer system in Fig. 3B

The experiment setup in Fig. 3 inherited the simulation setup in Fig. 2M, except that the load resistance *R_l_* was 2 kilohms. The neutral and load resonators resonated at 15 MHz by using a 216.5-pF capacitor (251R15S221JV4S, Johanson Technology). For the variable capacitor of the source resonator, NCD2400M with a capacitance range of 12.5 to 194 pF was used. To cover the wide capacitance range for the source-to-neutral coupling that matches the neutral-to-load coupling element-wise, two variable capacitors of GXD60000 (6 to 60 pF) and GXD13000 (2 to 13 pF) were used for the experiment. Last, the flipping of the coil was accomplished by using a 2.54-mm jumper socket to switch the connection of the coils.

### Tuning process of the system parameters

In the experiment of this work, the system parameters are tuned as follows. First, on the basis of the inductive coupling coefficients between the load and the neutrals obtained from the commercial electromagnetic simulator, we tune the flippable elements and the coupling capacitors between the source and neutral resonators to achieve μ*_i_* = κ*_i_*. Second, we digitally adjust the capacitor of the source resonator to fulfill the PT-symmetric conditions described in text S2. The code to control the variable capacitance using the Arduino is provided in the Data and materials availability statement. In the practical situations in which the coupling coefficients between neutrals and the load resonator are unknown in prior, the tuning can be done through numerical optimization. For example, if one disregards the coupling between the neutrals, then the maximization of the induced voltage by controlling the amplitudes of the multiple inputs can be modeled as a quadratic programming (*33*). The reduction of the design parameter dimension from *R* × *C^N^* in the conventional MISO system to *R*^*N+*1^ in the proposed system reduces the cost of the algorithm accordingly.

### Flexible receiver fabrication

The fabrication of the flexible receiver began with spin-coating and curing of the polyimide solution [poly(pyromellitic dianhydride-co-4,4′-oxydianiline), amic acid solution, Merck KGaA, Germany] on a silicon oxide wafer. Then, the first Cu layer was deposited (8 μm) for the flexible receiver coil and patterned by photolithography and wet etching. An epoxy layer (SU-8 10, MicroChem Corp., USA) was spin-coated and patterned via holes. The second Cu layer (8 μm) was deposited and patterned for chip pads. Another epoxy layer was spin-coated and patterned for encapsulation and contact pad opening. For the transfer of the fabricated coil, a polydimethylsiloxane (PDMS; SYLGARD 184, Dow Corning Corp., USA) stamp was attached to the antenna to pick up the device from the wafer. Then, a small amount of an optical adhesive was applied to the surface of the PDMS substrate to attach the antenna to the PDMS substrate. For soldering chips on the receiver, the exposed Cu pads were plated with Sn by applying an Sn plating solution [100 ml of deionized water, 0.5 g of Sn(II) chloride anhydrous (Alfa Aesar, USA), 2.0 g of thiourea (Alfa Aesar, USA), and 3.0 g of amidosulfonic acid (Alfa Aesar, USA)] for 60 s and were rinsed with water. After applying the solder paste (SID291SNL250T3, ChipQuik, USA) to the Sn-plated pads, chips such as the capacitor (ELE-C1005) and resistor (ELE-R1005F) were placed on the pads, and heat was applied with a heat gun to solder the chips.

### Experimental verification of flexible receivers

The experimental setup for flexible receivers inherited the setup for rigid circuits in Fig. 3, except that the load resistance *R_l_* was 4.7 kilohms. A Styrofoam spacer was placed between the neutrals and flexible receivers to define the distal displacement because the permittivity of Styrofoam (~1.1) is fairly similar to that of air (~1.0). The lateral displacement was measured between the center of a central neutral and the center of the load resonator. The response was measured at distal displacements of 7, 9.5, and 12 mm and lateral displacements from 0 to 50 mm in 5-mm increments. The bending radius of the flexible load resonator was controlled by attaching the device to the Styrofoam cylinders carved with the desired radii. The flexible resonator was attached to the cylinder by using single-sided 3M tape (fig. S10). The bending radius was controlled from 7 to 3 cm with a decrement of 1 cm. The rotation of the flexible load resonator was controlled by attaching the device to the Styrofoam holder adjusted on angular displacement of longitudinal, lateral, and distal direction (fig. S10, H to N, O to U, and V to AB). The rotation was controlled from 0° to 50°.

### Ex vivo experiment using a flexible receiver with an LED

The experimental setup for the ex vivo experiment was identical to the setup for rigid circuits in Fig. 3, except that the load resonator was replaced by the flexible load resonator and the porcine tissue was placed in between the resonator and neutrals. The distal displacement of the flexible load resonator was determined by stacking the porcine tissues with a thickness of 3 mm. The resonator was attached upside down conformally to the porcine tissue. The lateral displacement was measured from the center of the neutrals to the load resonator by using a ruler.

## Supplementary Materials

### This PDF file includes:

Supplementary Texts S1 to S3 Figs. S1 to S14
