# Supplementary material for: Wide-range robust wireless power transfer using heterogeneously coupled and flippable neutrals in parity-time symmetry
Source: Sci Adv. 2022 Jun 15;8(24):eabo4610. doi: 10.1126/sciadv.abo4610 (PMC9200287; doi:10.1126/sciadv.abo4610)
Supplement: Supplementary file 1 — Supplementary Texts S1 to S3 Figs. S1 to S14 [file sciadv.abo4610_sm.pdf]

Science Advances
AAAS

Supplementary Materials for

Wide-range robust wireless power transfer using heterogeneously coupled and flippable neutrals in parity-time symmetry

Hyunwoo Kim *et al.*

Corresponding author: Sanghoek Kim, sanghoek@khu.ac.kr; Dae-Hyeong Kim, dkim98@snu.ac.kr

Sci. Adv. **8**, eabo4610 (2022)
DOI: 10.1126/sciadv.abo4610

This PDF file includes:

Supplementary Texts S1 to S3
Figs. S1 to S14

Supplementary Text

Text S1. Hamiltonian of the oligomer WPT system

Based on the coupled-mode theory, we demonstrate that the WPT system having multiple neutrals with similar resonant frequencies have the Hamiltonian expressed as the equation (3). The expressions for various parameters, including the gain rate g_s, the loss rate γ'_l, and the frequency offset $\Delta\omega_m$, are obtained in terms of the circuit components for the configurations of homogeneous (Fig. 2I) and heterogeneous (Fig. 2M) coupling.

For the purpose of explanation, the circuit schematics of Fig. 2I and M are redrawn in Fig. S2A and B with the proper labeling of circuit components, voltages, and currents. The gain element of the source is represented by the negative resistance $-R_s$. As mentioned in the main text, the alphabetical subscripts (*s, l*) indicate the source and the load and the numerical subscript i indicates the *i*-th neutral unit. For example, $V_m(m = s, l, 1,2, \ldots, N)$ refers to the voltage amplitude of each resonator and I_m refers to the current flowing through the inductor of each resonator.

Considering the inductive coupling between the resonators, the voltages and the currents are related as

$$\boldsymbol{V} = i\omega \begin{bmatrix} L_s & -M_{s1} & \cdots & -M_{sN} & 0 \\ -M_{1s} & L_1 & \cdots & -M_{1N} & -M_{1l} \\ \vdots & \vdots & \ddots & \vdots & \vdots \\ -M_{Ns} & -M_{N1} & \ldots & L_N & -M_{Nl} \\ 0 & -M_{l1} & \cdots & -M_{lN} & L_l \end{bmatrix} \boldsymbol{I}, \tag{S1}$$

where $\boldsymbol{V} = [V_s, V_1, V_2, \ldots, V_N, V_l]^t$ and $\boldsymbol{I} = [I_s, I_1, I_2, \ldots, I_N, I_l]^t$, and M_{mn} $(m, n = s, l, 1,2, \ldots, N)$ is the mutual inductance between the *m*-th and *n*-th resonators. In the heterogeneously coupled configuration (Fig. 2M or Fig. S2B), the source-to-neutral mutual inductances M_{is} $(i = 1,2, \ldots, N)$ are zero, but instead they are capacitively coupled. Taking the inverse of (S1), we have $i\omega\boldsymbol{I} = [\boldsymbol{Y}]\boldsymbol{V}$, where $[\boldsymbol{Y}]$ is the admittance matrix between $\boldsymbol{I}$ and $\boldsymbol{V}$.

(a) Homogeneously coupled configuration

Figure S2A shows the circuit schematic in which the source-to-neutral couplings are inductive like the neutral-to-load couplings. Applying the Kirchhoff's voltage law at each node of the resonator in Fig. S1A yields

$$\begin{aligned} -\omega^2 C_s V_s - \frac{i\omega V_s}{R_s} + i\omega I_s &= 0 \\ -\omega^2 C_1 V_1 + i\omega I_1 &= 0 \\ &\vdots \\ -\omega^2 C_N V_N + i\omega I_N &= 0 \\ -\omega^2 C_l V_l + \frac{i\omega V_l}{R_l} + i\omega I_l &= 0. \end{aligned} \tag{S2}$$

Substituting the $i\omega\boldsymbol{I} = [\boldsymbol{Y}]\boldsymbol{V}$ into (S2) and dividing each row by the corresponding capacitance $2\omega C_m$ gives

$$
0 = \begin{bmatrix} -\frac{\omega}{2} - \frac{i}{2R_sC_s} + \frac{Y_{ss}}{2\omega C_s} & \frac{Y_{s1}}{2\omega C_s} & \cdots & \frac{Y_{s2}}{2\omega C_s} & \frac{Y_{sl}}{2\omega C_s} \\ \frac{Y_{1s}}{2\omega C_0} & -\frac{\omega}{2} + \frac{Y_{11}}{2\omega C_0} & \cdots & \frac{Y_{1N}}{2\omega C_0} & \frac{Y_{1l}}{2\omega C_0} \\ \vdots & \vdots & \ddots & \vdots & \vdots \\ \frac{Y_{Ns}}{2\omega C_0} & \frac{Y_{N1}}{2\omega C_0} & \cdots & -\frac{\omega}{2} + \frac{Y_{NN}}{2\omega C_0} & \frac{Y_{Nl}}{2\omega C_0} \\ \frac{Y_{ls}}{2\omega C_0} & \frac{Y_{lNl1}}{2\omega C_0} & \cdots & \frac{Y_{lN}}{2\omega C_0} & -\frac{\omega}{2} + \frac{i}{2R_lC_0} + \frac{Y_{ll}}{2\omega C_0} \end{bmatrix} \begin{bmatrix} V_s \\ V_1 \\ \vdots \\ V_N \\ V_l \end{bmatrix}, \quad \text{(S3)}
$$

where $C_0 = C_1 = \cdots = C_N = C_l$. Using the approximation $Y_{mm} \approx 1/L_m$ and $Y_{sl} = Y_{ls} \approx 0$ and $\frac{1}{2\omega L_m C_m} - \frac{\omega}{2} = \frac{1}{2\omega}({\omega_m}^2 - \omega^2) \approx (\omega_m - \omega)$, (S3) reduces to the form of the equation (3) with the gain rate $g_s = 1/(2R_sC_s)$, the output loss rate $\gamma_l' = 1/(2R_lC_l)$, the source-to-neutral coupling rate $\mu_i = Y_{is}/(2\omega C_0)$, the load-to-neutral coupling rate $\kappa_i = Y_{il}/(2\omega C_0)$, and the coupling rate between the neutrals $\kappa_{ij} = Y_{ij}/(2\omega C_0)$, while the frequency offset $\Delta\omega_m = 0$. When *N*=0, the expression for the coupled-mode parameters of the oligomer system are consistent with the one for the dimer system in (*1, 8, 9, 28, 30, 37*).

(b) Heterogeneously coupled configuration

In Fig. S2B, the source-to-neutral couplings are capacitive, while the neutral-to-load couplings are inductive. In this heterogeneously coupled configuration, since the source resonators are not inductively coupled to any other resonators ($M_{ms} = 0$ for all $m = 1,2,\ldots,N,l$), the admittance element Y_{ms} are zero for all m ($=1,2,\ldots,N,l$). The circuit equations by the Kirchhoff's voltage law are

$$
\begin{aligned}
-\omega^2 C_s V_s - \textstyle\sum_{i=1}^{N} \omega^2 C_{si}(V_s - V_i) - \frac{i\omega V_s}{R_s} + i\omega I_s &= 0 \\
-\omega^2 C_1 V_1 - \omega^2 C_{s1}(V_1 - V_s) + i\omega I_1 &= 0 \\
&\vdots \\
-\omega^2 C_N V_N - \omega^2 C_{sN}(V_N - V_s) + i\omega I_N &= 0 \\
-\omega^2 C_l V_l + \frac{i\omega V_l}{R_l} + i\omega I_l &= 0
\end{aligned} \quad \text{(S4)}
$$

We substitute the $i\omega \boldsymbol{I} = [\boldsymbol{Y}]\boldsymbol{V}$ into (S4) and divide each row by the corresponding capacitance $2\omega C_m$ to have

$$
0 = \begin{bmatrix} -\frac{\omega}{2C_s}\left(C_s + \sum_{i=1}^{N} C_{si}\right) - \frac{i}{2R_sC_s} + \frac{Y_{ss}}{2\omega C_s} & \frac{\omega C_{s1}}{2C_s} & \cdots & \frac{\omega C_{sN}}{2C_s} & 0 \\ \frac{\omega C_{s1}}{2C_0} & -\frac{\omega}{2}\left(1 + \frac{C_{s1}}{C_0}\right) + \frac{Y_{11}}{2\omega C_0} & \cdots & \frac{Y_{1N}}{2\omega C_0} & \frac{Y_{1l}}{2\omega C_0} \\ \vdots & \vdots & \ddots & \vdots & \vdots \\ \frac{\omega C_{sN}}{2C_0} & \frac{Y_{N1}}{2\omega C_0} & \cdots & -\frac{\omega}{2}\left(1 + \frac{C_{sN}}{C_0}\right) + \frac{Y_{NN}}{2\omega C_0} & \frac{Y_{Nl}}{2\omega C_0} \\ 0 & \frac{Y_{l1}}{2\omega C_0} & \cdots & \frac{Y_{lN}}{2\omega C_0} & -\frac{\omega}{2} + \frac{i}{2R_lC_l} + \frac{Y_{ll}}{2\omega C_0} \end{bmatrix} \begin{bmatrix} V_s \\ V_1 \\ \vdots \\ V_N \\ V_l \end{bmatrix} \quad \text{(S5)}
$$

Taking the approximation $Y_{mm} \approx 1/L_m$ and $\frac{1}{2\omega L_m C_m} - \frac{\omega}{2} = \frac{1}{2\omega}({\omega_m}^2 - \omega^2) \approx (\omega_m - \omega)$ again transforms (S5) into the form of the equation (3) with the gain rate $g_s = 1/(2R_sC_s)$, the output loss rate $\gamma_l' = 1/(2R_lC_l)$, the source-to-neutral coupling rate $\mu_i = \omega C_{si}/(2C_0)$, the load-to-neutral coupling rate $\kappa_i = Y_{il}/(2\omega C_0)$, the coupling rate between the neutrals $\kappa_{ij} = Y_{ij}/(2\omega C_0)$. Notice that in the heterogeneous configuration, the frequency offset $\Delta\omega_m$ are not zero as $\Delta\omega_s = \sum_{i=1}^{N} \mu_i$, $\Delta\omega_i = \mu_i$ for $(i = 1, \dots, N)$, and $\Delta\omega_l = 0$.

<u>Text S2. The conditions for PT symmetry</u>

For a system to be PT symmetric, $PT\hat{H}\psi = \hat{H}PT\psi$ must be satisfied for any state ψ. In our oligomer system, the operator P is the parity transformation between the source and the load ($s \leftrightarrow l$) and T is the time reversal operation which can be effectively made by taking the complex conjugate ($i \rightarrow -i$). Explicitly, for a given Hamiltonian H in the equation (3),

$$PTH\psi = \begin{bmatrix} 0 & -\kappa_1 & -\kappa_2 & & -\kappa_N & -(\omega_l - \Delta\omega_l) + i\gamma'_l \\ -\mu_1 & -(\omega_1 - \Delta\omega_1) & -\kappa_{12} & \cdots & -\kappa_{1N} & -\kappa_1 \\ -\mu_2 & -\kappa_{12} & -(\omega_2 - \Delta\omega_2) & & -\kappa_{2N} & -\kappa_2 \\ \vdots & \vdots & & \ddots & \vdots & \\ -\mu_N & -\kappa_{1N} & -\kappa_{2N} & \cdots & -(\omega_N - \Delta\omega_N) & -\kappa_N \\ -(\omega_s - \Delta\omega_s) - ig_s & -\mu_1 & -\mu_2 & & -\mu_N & 0 \end{bmatrix} \begin{bmatrix} V_s^* \\ V_1^* \\ V_2^* \\ \vdots \\ V_N^* \\ V_l^* \end{bmatrix}, \quad \text{(S6)}$$

where a* indicates the complex conjugate of a. For the PT symmetry to hold, the above should be equal to

$$HPT\psi = \begin{bmatrix} -(\omega_s - \Delta\omega_s) + ig_s & -\mu_1 & -\mu_2 & & -\mu_N & 0 \\ -\mu_1 & -(\omega_1 - \Delta\omega_1) & -\kappa_{12} & \cdots & -\kappa_{1N} & -\kappa_1 \\ -\mu_2 & -\kappa_{12} & -(\omega_2 - \Delta\omega_2) & & -\kappa_{2N} & -\kappa_2 \\ \vdots & \vdots & & \ddots & \vdots & \\ -\mu_N & -\kappa_{1N} & -\kappa_{2N} & \cdots & -(\omega_N - \Delta\omega_N) & -\kappa_N \\ 0 & -\kappa_1 & -\kappa_2 & & -\kappa_N & -(\omega_l - \Delta\omega_l) - i\gamma'_l \end{bmatrix} \begin{bmatrix} V_l^* \\ V_1^* \\ V_2^* \\ \vdots \\ V_N^* \\ V_s^* \end{bmatrix} \quad \text{(S7)}$$

for any state $\boldsymbol{V} = [V_s, V_1, V_2, \ldots, V_N, V_l]^t$.

Comparison between (S6) and (S7) concludes that the conditions for the PT symmetry are as follows:

1. $\mu_i = \kappa_i$, where $i = 1, 2, \ldots, N$
2. $\omega_l - \Delta\omega_l = \omega_s - \Delta\omega_s$
3. $g_s = \gamma'_l$

The first condition is fulfilled by adjusting the source-to-neutral coupling rate to the neutral-to-load coupling rate including the signs, which requires a mechanically controlled source positioner (variable coupling capacitors and the flipping switches) for the homogeneously-(heterogeneously-)coupled configuration. The second condition is inherently satisfied for the homogeneously coupled configuration, since the frequency offsets $\Delta\omega_m$ are all zero. For the heterogeneously coupled configuration, however, the source capacitor C_s needs to be adjusted to match $\omega_s = \frac{1}{\sqrt{L_s C_s}} = \omega_l + \sum_{i=1}^{N} \mu_i$. In other words, the self-resonant frequency of the source resonator should be set different from the other resonators ($\omega_1 = \cdots = \omega_l = \omega_0$) by the coupling rates to achieve the PT symmetry. It requires the source capacitor to be controllable as well, leading the total dimension of design spaces to be R^{N+1}. The third condition can be satisfied if the gain g_s of the source saturates to γ_l' at steady state by using a non-linear amplifier (*8, 9, 13, 19, 20*).

Text S3. Eigenvalues and eigenstates for the Hamiltonian with a variable gain and the decision of the saturated gain

To find the eigenvalues, we assume $\mathbf{V} \propto e^{i\omega t}$ and obtain the characteristic equation of H.

$$Det(H+\omega I)$$
$$= Det\begin{bmatrix} -(\omega_0-\omega)+ig_s & -\mu_1 & \cdots & -\mu_N & 0 \\ -\mu_1 & -(\omega_0-\omega) & \cdots & -\kappa_{1N} & -\kappa_1 \\ -\mu_2 & -\kappa_{21} & \cdots & -\kappa_{2N} & -\kappa_2 \\ \vdots & \vdots & \ddots & \vdots & \vdots \\ -\mu_N & -\kappa_{N1} & \cdots & -(\omega_0-\omega) & -\kappa_N \\ 0 & -\kappa_1 & \cdots & -\kappa_N & -(\omega_0-\omega)-i\gamma'_l \end{bmatrix} = 0 \qquad \text{(S8)}$$

Note that all the resonators are assumed to have the identical resonant frequency $\omega_m - \Delta\omega_m = \omega_0$ $(m = s, l, 1,2,\ldots,N)$. The roots of the characteristic equation are the eigenvalues of the Hamiltonian. At steady state, the oligomer WPT system would operate at the eigenstate **V** and real eigenvalue ω which requires the lowest gain $g_s = g_{s,sat}$. To obtain real-valued ω, we separate the real and the imaginary part of (S8). Specifically, the imaginary part of (S8) has a relatively simple form of

$$0 = g_s[p_{N+1}(\omega_0-\omega)^{N+1}+\ldots+p_0] - \gamma'_l[p_{N+1}(\omega_0-\omega)^{N+1}+\ldots+p_0], \qquad \text{(S9)}$$

where p_j indicates the *j*-th coefficient of an (*N+1*)-th order polynomial. This can be regarded as a generalized expression of the imaginary part of the determinant in the dimer configuration where *N*=0 (*8, 9, 13*). In PT–symmetric regime where $g_{s,sat} = \gamma'_l$, the equation (S9) is naturally satisfied. The eigenvalues can be obtained by finding zeros of the real part of (S8) with substituting $g_s = \gamma'_l$. The operation of the oligomer system at these modes makes the system PT symmetric.

If, however, g_s smaller than γ'_l that allows real eigenfrequencies exists, the oligomer would not operate at a PT–symmetric mode. The value of g_s other than γ'_l yielding real eigenfrequencies can be found in a similar manner as in (*8*). First, the real-valued roots $\Delta\omega = \omega_0 - \omega$ of the polynomial in the bracket of (S9) are found. For each value of such roots, the gain of the source g_s can be selected to make the real part of the determinant (S8) zero as well. Through this, we obtain pairs of g_s and real eigenvalues that can possibly be the operating mode of the oligomer system.

The obtained g_s with respect to displacements are plotted in Fig. S4A for homogeneously coupled and Fig. S4B for heterogeneously coupled configurations. Both plots show that for the displacement smaller than ~30 mm, the lowest gain with real eigenvalues is γ'_l. Within this region, the oligomer operates PT symmetrically. Beyond the region, the gain lower than γ'_l to yield a real eigenfrequency exists. As the non-linear amplifier of the oligomer system saturates to the gain $g_{s,sat} < \gamma'_l$, the PT symmetry does not hold any more. The eigenvalues plotted in Fig. 2K, O, and 3C are the ones that correspond to the saturated gain at each displacement. In other words, for the PT–symmetric region (*x* = 0 ~ 30 mm), the eigenfrequencies are obtained with the gain $g_s = \gamma'_l$, while for the region beyond it, they correspond to the gain $g_s < \gamma'_l$.

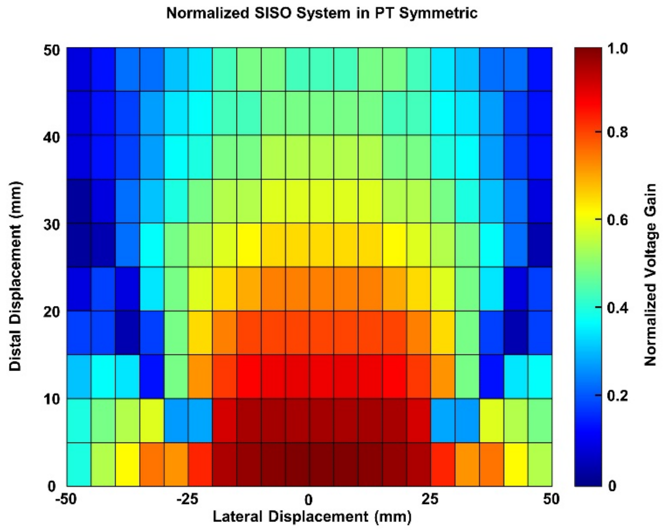

Fig. S1. The normalized voltage gain for the Single-Input Single-Output (SISO) PT–symmetric system with unbalanced coil sizes. In this plot, the voltage gain of Fig. 1E is normalized to its maximum value to clearly visualize its degradation with the displacement. The dimension of unbalanced source coil for the SISO system is 30 × 60 mm^2, which is the same as the aperture of the three neutrals for Fig. 1F. To build a PT−symmetric dimer system, in which the resonant frequency of the source is equal to the load, the capacitor at the source is set to have a lower capacitance than that of the load.

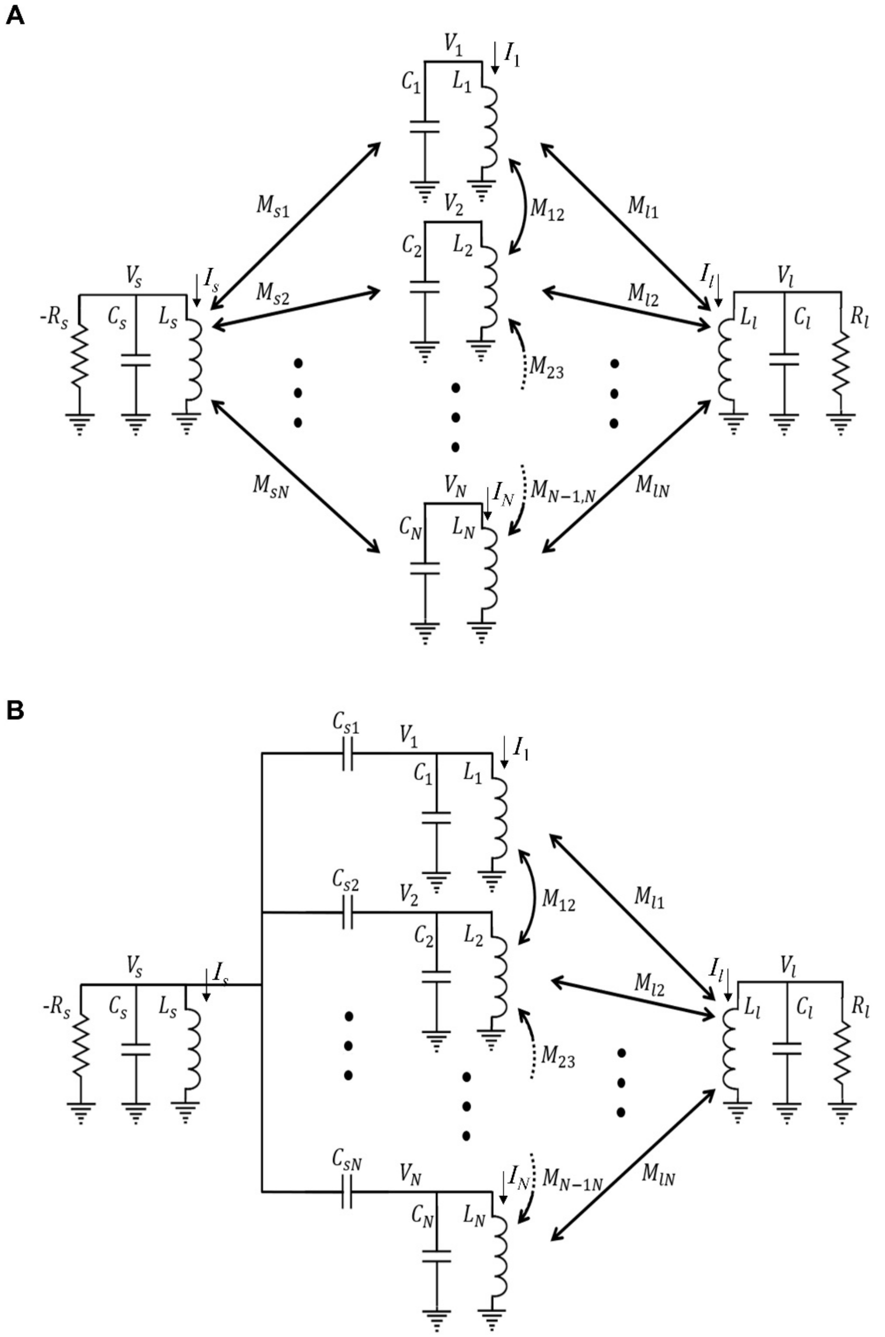

Fig. S2. Circuit diagram used to derive the dynamic equations of the oligomer system. (**A**) Circuit schematic of the homogeneously-coupled oligomer system. The source and neutrals are inductively coupled like the neutrals and loads. (**B**) Circuit schematic of the heterogeneously-coupled oligomer system. The source and neutrals are capacitively coupled, whereas the neutrals and load are inductively coupled.

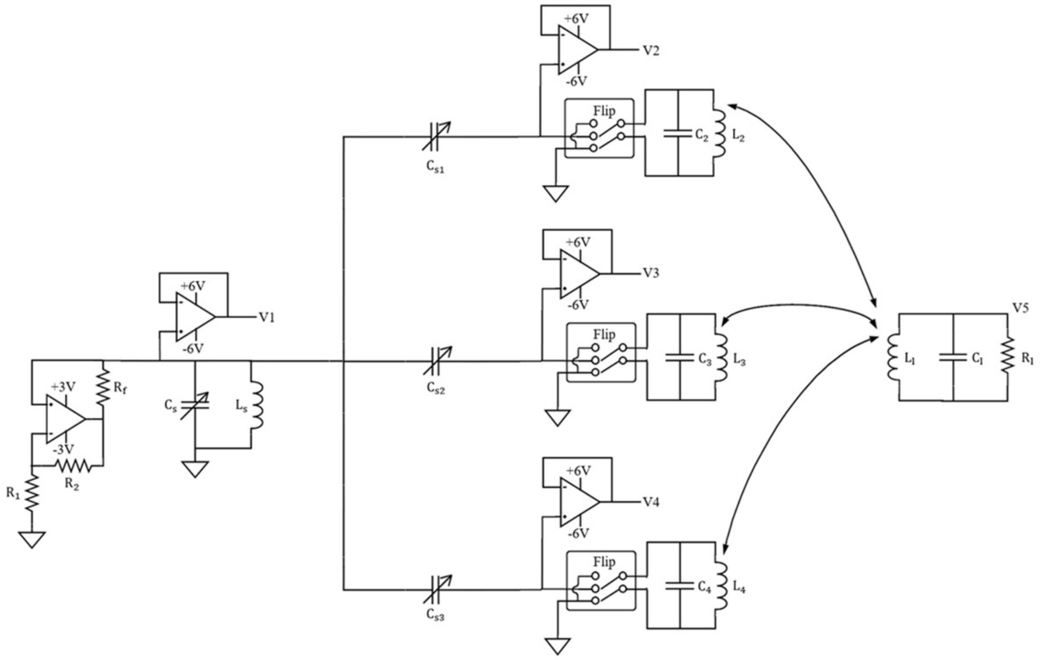

Fig. S3. Circuit diagram of the PT–symmetric oligomer system with flips. The source and neutrals are capacitively coupled via variable capacitors to improve the practicality of the overall system. The coupling level of capacitors can be easily controlled in accordance with the variation of the inductive coupling between the neutrals and load.

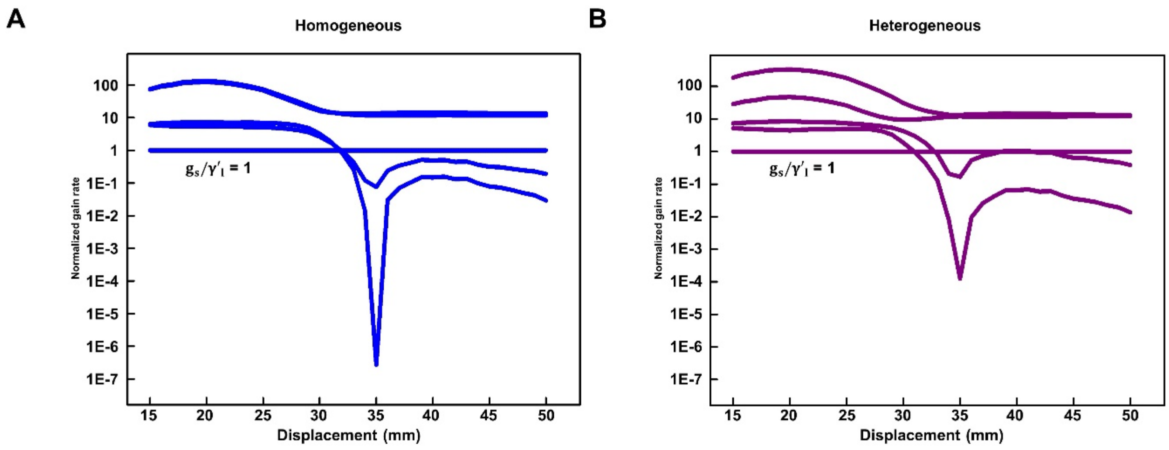

Fig. S4. Gain rate g_s with real eigenvalues normalized by the loss rate γ_l' at the load. Gain rates for (**A**) the homogenous configuration (Fig. 2I) and (**B**) the heterogeneous configuration (Fig. 2M). For both configurations, up to a displacement of ~30 mm, the lowest gain with real eigenvalues is equal to γ_l'. Within this region, the gain saturates to γ_l', leading the system to be PT–symmetric ($g_{s,sat} = \gamma_l'$). Beyond the region, there exists a lower gain than γ_l' that has real eigenvalues. The system reaches a steady state that is not PT–symmetric ($g_{s,sat} < \gamma_l'$).

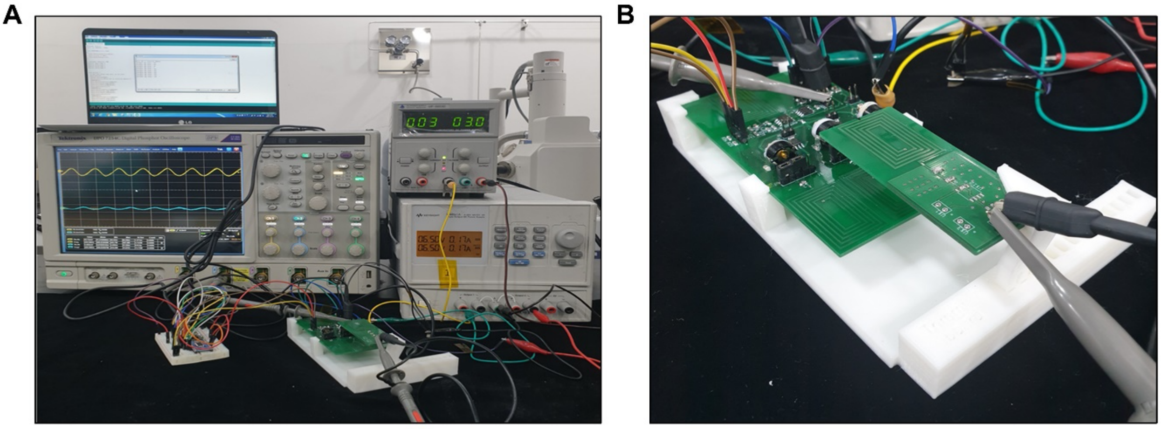

Fig. S5. Photograph of the experimental setup of heterogeneously coupled and flippable neutral units in the PT–symmetric pentamer system. (**A**) Photograph of the experimental setup of the prototype device. (**B**) Magnified view of the measurement setup. The distal and lateral displacement are determined by the custom-made 3D-printed holder.

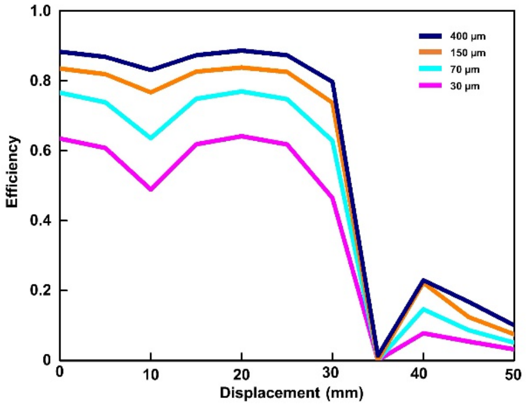

Fig. S6. Efficiency of the higher-order PT–symmetric pentamer system (simulation result) for various coil thicknesses. The simulation result shows that the thicker coil yields the higher efficiency. The distal distance between the neutral layer and the load is fixed as 7 mm, and the load impedance is 1 kΩ.

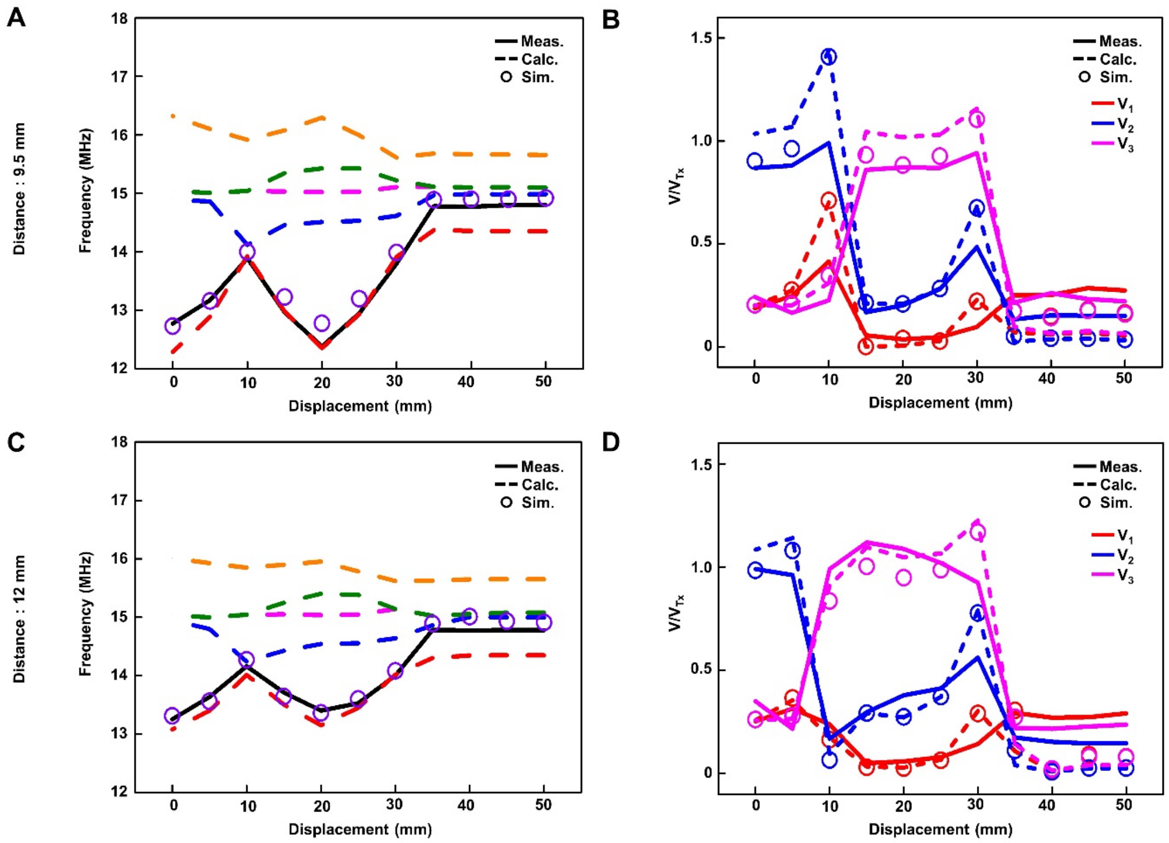

Fig. S7. Experimental demonstration of the heterogeneously-coupled PT–symmetric pentamer system at different distal displacement between the neutrals and load. (A–B), Eigenvalues of the system and voltage amplitudes of the neutrals at a distal displacement of 9.5 mm. (**A**) Multiple eigenvalues versus lateral displacement in the pentamer system. An eigenvalue chosen as the operating frequency of the system appeared in the measurements, as indicated by the black solid line. (**B**) Measured voltage amplitudes of the three neutrals arranged between the source and load. The results resemble the calculated and simulated data fairly closely. **(C–D),** Eigenvalues of the system and voltage amplitudes of the neutrals at a distal displacement of 12 mm. (**C**) Multiple eigenvalues versus lateral displacement in the pentamer system. An eigenvalue chosen as the operating frequency of the system appeared in the measurements, as indicated by the black solid line. (**D**) Measured voltage amplitudes of the three neutrals arranged between the source and load. The results resemble the calculated and simulated data fairly closely.

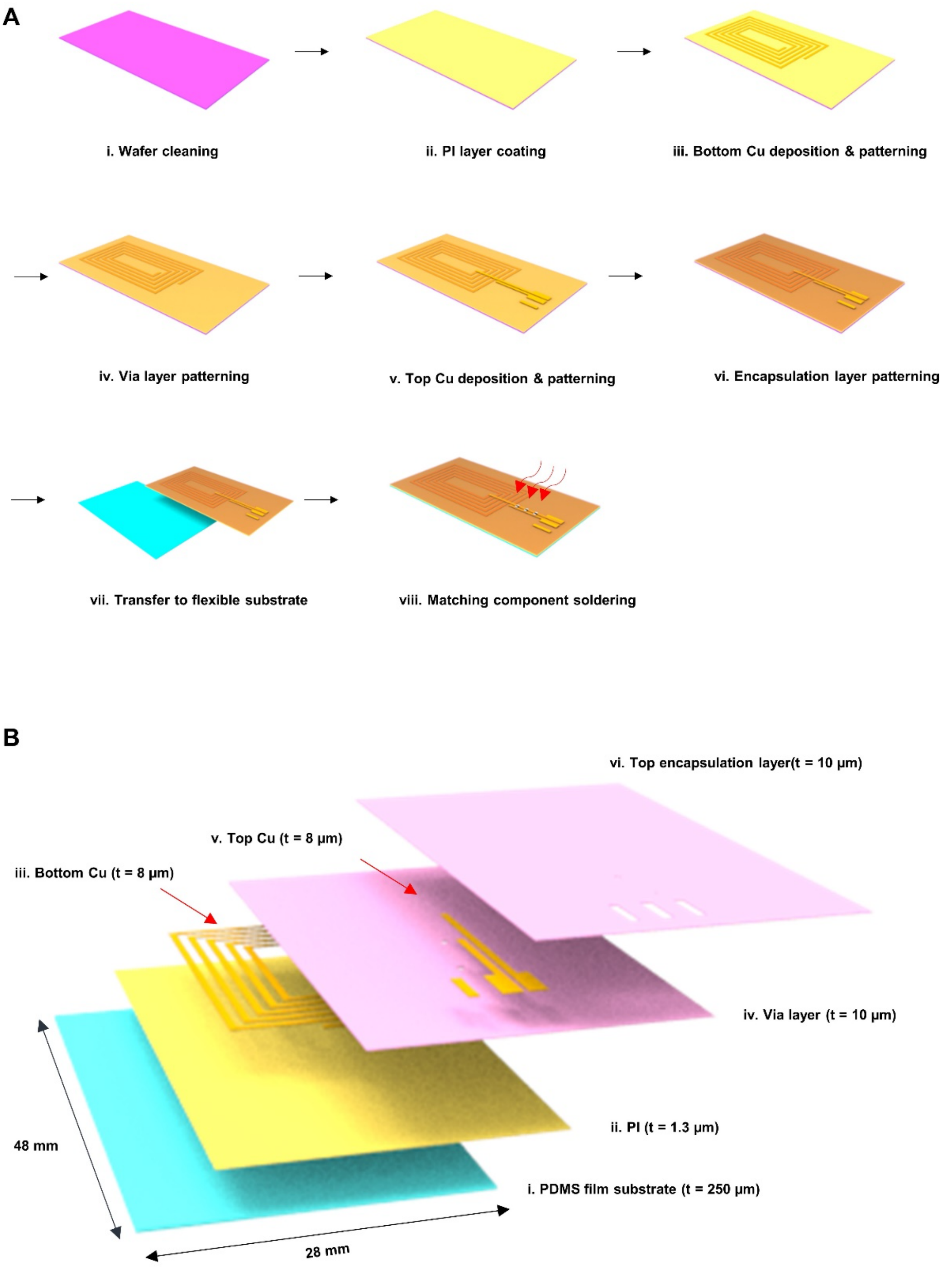

Fig. S8. Fabrication steps and exploded view of the flexible load resonator. (A) Fabrication step. (**i**) Cleaning of the handling wafer. (**ii**) Spin-coating of a PI layer on the wafer. (**iii**) Photolithography and etching of the bottom Cu layer for coil patterning. (**iv**) Photolithography of the bottom epoxy (SU-8) layer for patterning of the via hole. (**v**) Photolithography and etching of the top Cu layer for bridge patterning. (**vi**) Photolithography of the top epoxy (SU-8) layer for encapsulation and pad opening. (**vii**) Transfer of the fabricated load resonator from the wafer to a flexible substrate. (**viii**) Soldering of the matching component onto the pad opening by using soldering paste and applying heat. **(B)** Exploded view (**i**) PDMS film

substrate casted with the thickness of 250 μm (**ii**) PI was spin-coated with the thickness of 1.3 μm. (**iii**) Bottom Cu layer was deposited with the thickness of 8 μm. (**iv**) Via layer (Su-8 10) was spin-coated with the thickness of 10 μm. (**v**) Top Cu layer was deposited with the thickness of 8 μm. (**vi**) Top encapsulation layer (Su-8 10) was spin-coated with the thickness of 10 μm.

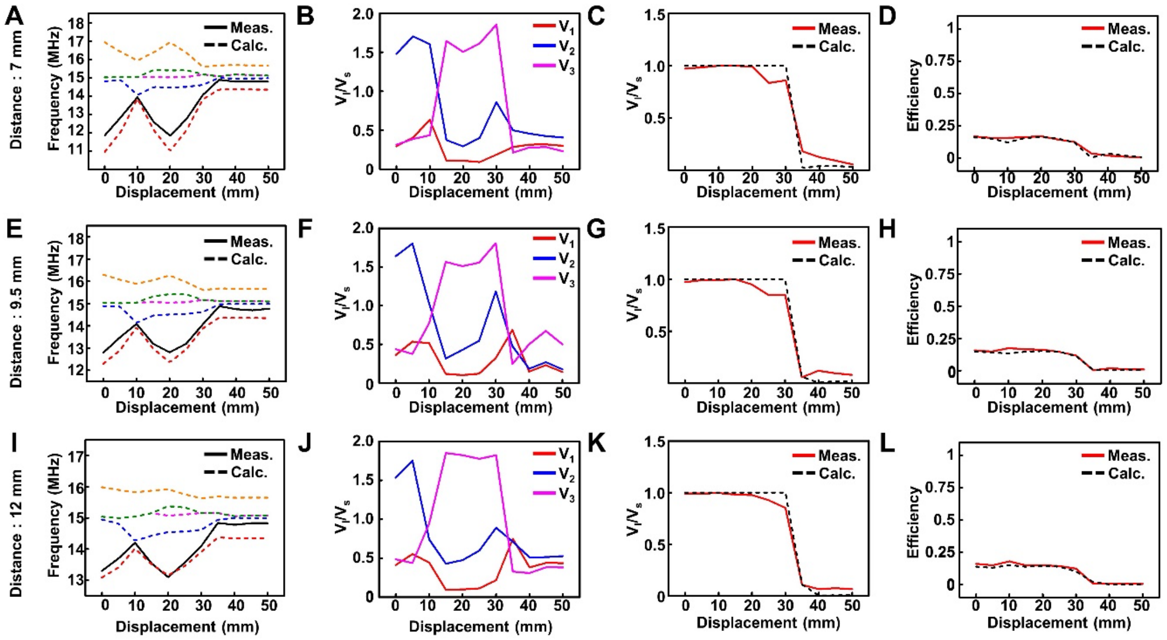

Fig. S9. System response of the PT–symmetric pentamer system using the flexible load resonator at different distal and lateral displacements. System response at distal displacements of (**A**–**D**), 7 mm; (**E**–**H**), 9.5 mm; and (**I**–**L**), 12 mm. (**A, E, I**) Five different eigenfrequencies versus lateral displacement in the pentamer system. The operating frequency of the system is indicated by a black solid line. (**B, F, J**) Voltage amplitudes of the three neutrals. (**C, G, K**) Measured and simulated voltage gain acquired from the pentamer system. The voltage gain is fairly constant across ~30 mm of lateral displacement. (**D, H, I**) Measured and simulated efficiencies acquired from the pentamer system. The efficiency is fairly constant across ~30 mm of lateral displacement.

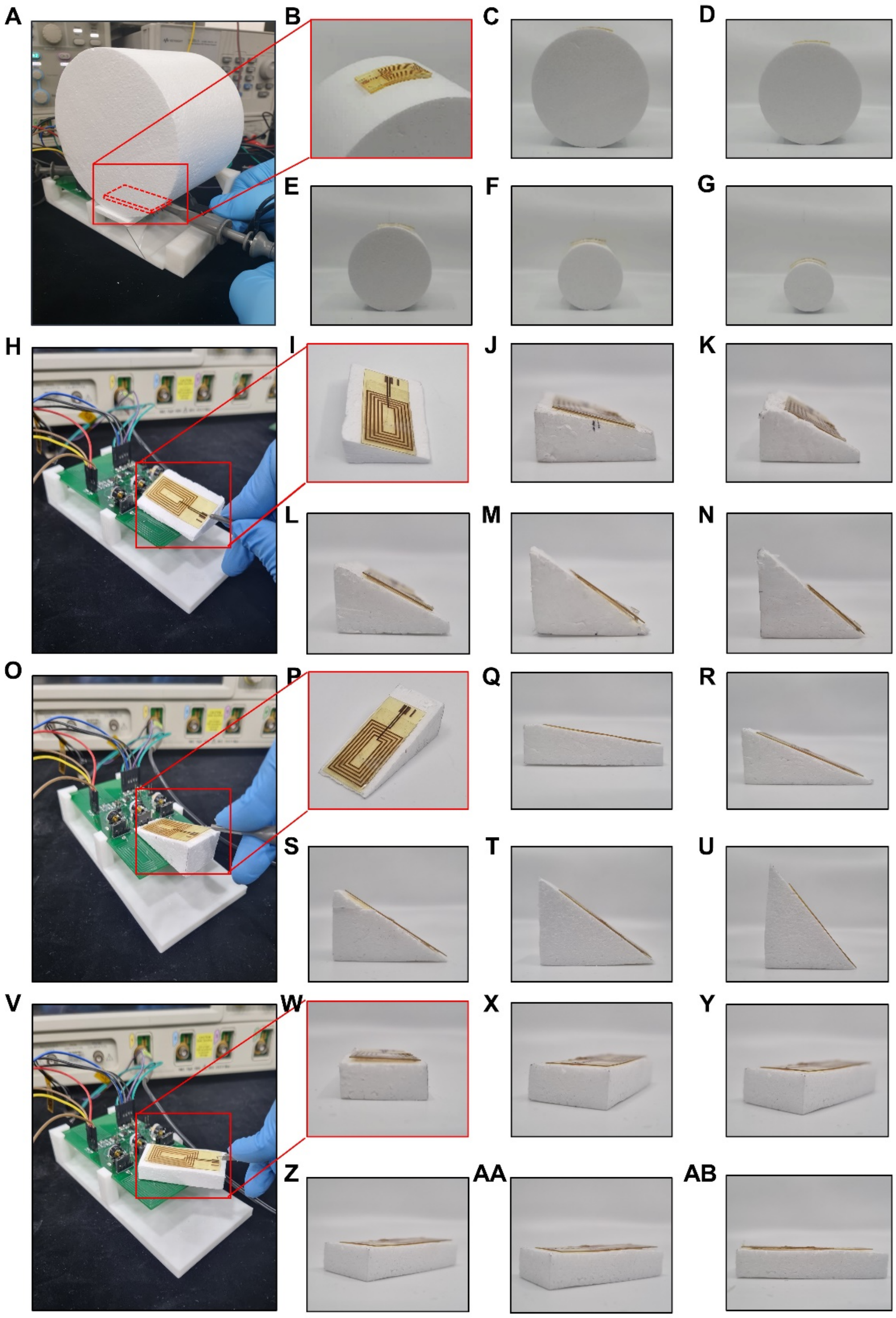

Fig. S10. Photographs of the experiments to characterize the effect of the flexural deformations and positional changes of the flexible load resonator. A flexible load resonator was conformally attached onto the Styrofoam structures for the voltage gain measurement. (**A, B**) Voltage gain measurement under the bending deformation of the load resonator with the bending radius of (**C**) 7 cm, (**D**) 6 cm, (**E**) 5 cm, (**F**) 4 cm, and (**G**) 3 cm. (**H, I**) Voltage gain measurement at the rotated position in the longitudinal direction with the

rotation angle of (**J**) 10°, (**K**) 20°, (**L**) 30°, (**M**) 40°, and (**N**) 50°. (**O, P**) Voltage gain measurement at the rotated position in the lateral direction with the rotation angle of (**Q**) 10°, (**R**) 20°, (**S**) 30°, (**T**) 40°, and (**U**) 50°. (**V, W**) Voltage gain measurement at the rotated position in the distal direction with the rotation angle of (**X**) 10°, (**Y**) 20°, (**Z**) 30°, (**AA**) 40°, and (**AB**) 50°.

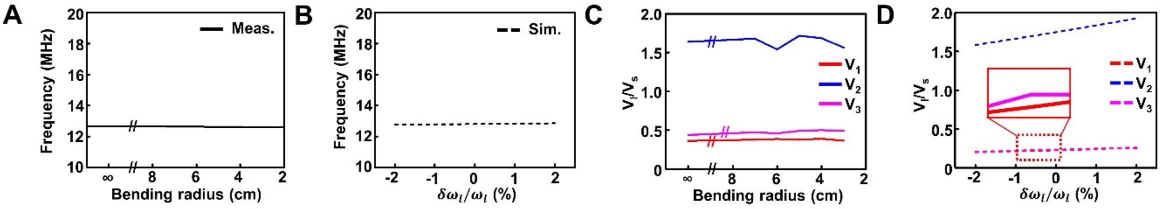

Fig. S11. System response of the PT–symmetric pentamer system using the flexible load resonator at various bending radii. (**A**) Measured operating frequency of the pentamer system at bending radii of 7, 6, 5, 4, and 3 cm. (**B**) Simulated operating frequency of the pentamer system as a function of the self-resonant frequency change of the flexible load resonator. The change in the eigenfrequency is negligible throughout the variations (**C**) Measured voltage amplitudes of three neutrals in the pentamer system at bending radii of 7, 6, 5, 4, and 3 cm. (**D**) Simulated voltage amplitudes of the three neutrals as a function of the self-resonant frequency change of the flexible load resonator. All the neutrals, including the center one, which couples the most strongly with the load, exhibit little change in amplitudes throughout the variations.

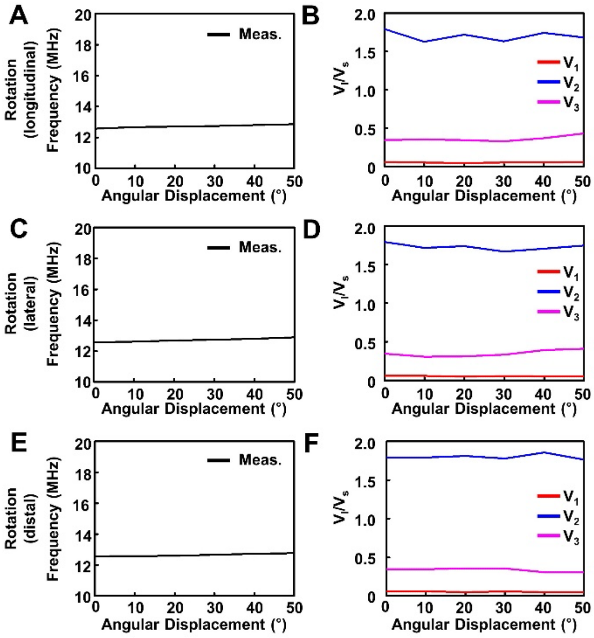

Fig. S12. Characterization of the PT–symmetric pentamer system with the flexible load resonator under various rotation conditions. (**A, C, E**) Operating frequency of the pentamer system at the angular displacement of 0°, 10°, 20°, 30°, 40°, and 50° in the longitudinal, lateral, and distal direction, respectively. (**B, D, F**) Voltage amplitudes of three neutrals in the pentamer system for the angular displacements of 0°, 10°, 20°, 30°, 40°, and 50° in the longitudinal, lateral, and distal direction, respectively.

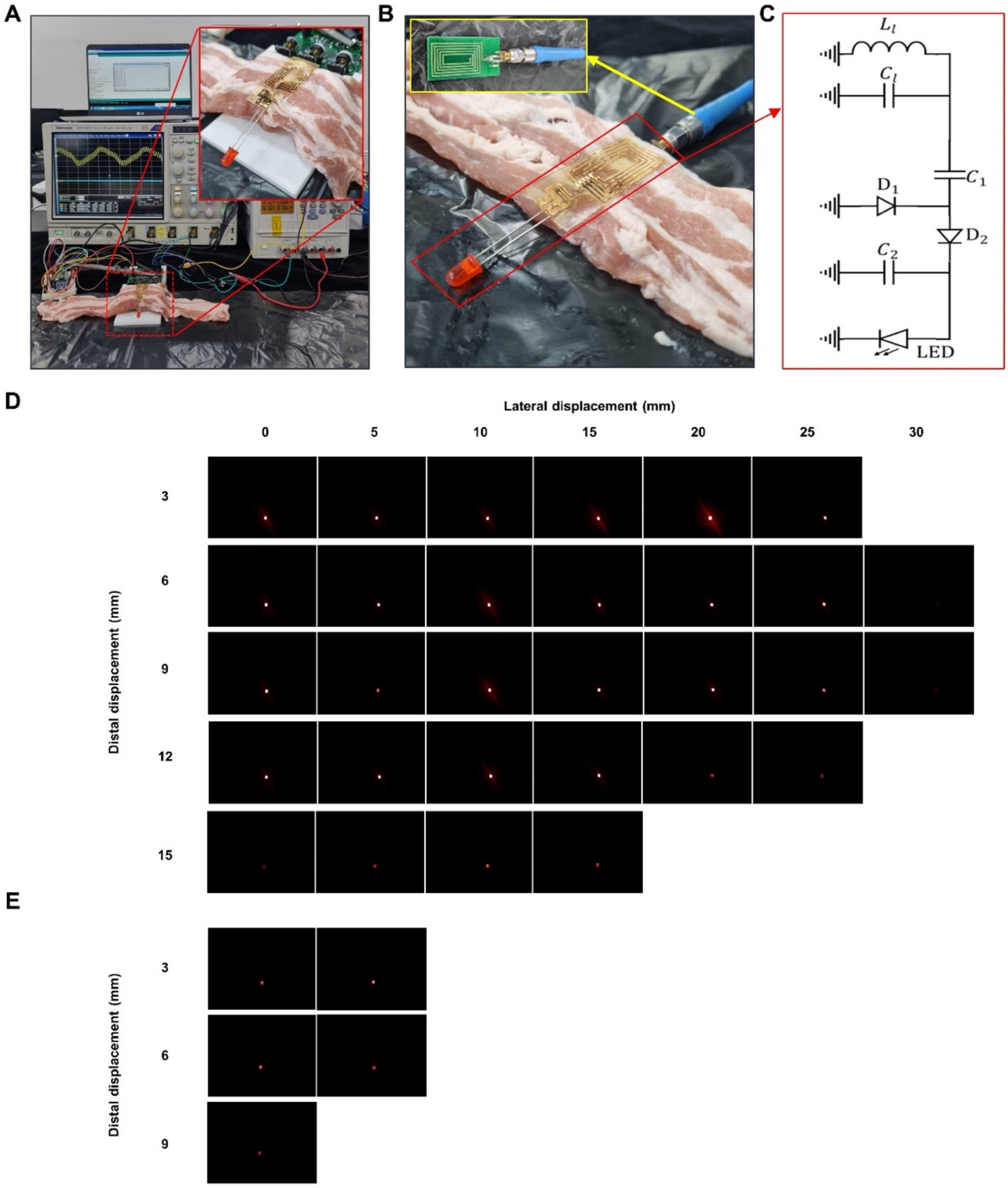

Fig. S13. Experimental setup for the *ex vivo* demonstration and the WPT test results. Experimental setup for the *ex vivo* demonstration of (**A**) heterogeneously coupled flappable PT–symmetric pentamer system and (**B**) conventional inductively-coupled WPT system. The load resonator was placed on the surface of the porcine tissue (3 mm) placed between the load and neutrals. An LED was used to show the reliable WPT through the porcine tissue. (**C**) The circuit diagram of the flexible load resonator with an LED for the *ex vivo* demonstration. Several components (capacitor, resistor, and rectifier) were added to convert AC voltage into DC voltage for the LED. (**D-E**) Images of the lightened LED at various distal and lateral displacements of (**D**) the PT–symmetric pentamer WPT system and (**E**) the conventional WPT system.

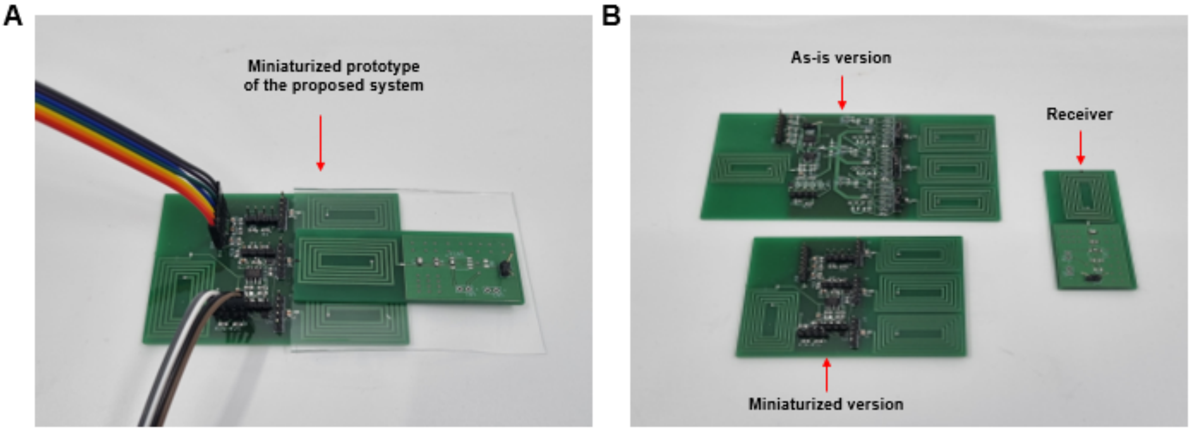

Fig. S14. The miniaturized prototype compared with the as-is prototype. (**A**) Image of a prototype for the miniaturized version of the system. (**B**) Comparison of the miniaturized and the as-is version. In the miniaturized version, the buffers are removed and the coupling capacitors between the source and neutral resonators are replaced by electronically-tunable capacitors that have a smaller size than the manually-tunable ones. It can reduce the system size from 113.7 × 66.3 mm^2 to 77 × 60.4 mm^2, which better meets the needs of the compact size for the contemporary wearable devices.
